# VEGF-B-induced vascular growth leads to metabolic reprogramming and ischemia resistance in the heart

**DOI:** 10.1002/emmm.201303147

**Published:** 2014-01-21

**Authors:** Riikka Kivelä, Maija Bry, Marius R Robciuc, Markus Räsänen, Miia Taavitsainen, Johanna MU Silvola, Antti Saraste, Juha J Hulmi, Andrey Anisimov, Mikko I Mäyränpää, Jan H Lindeman, Lauri Eklund, Sanna Hellberg, Ruslan Hlushchuk, Zhen W Zhuang, Michael Simons, Valentin Djonov, Juhani Knuuti, Eero Mervaala, Kari Alitalo

**Affiliations:** 1 Wihuri Research Institute and Translational Cancer Biology Research Program, University of HelsinkiHelsinki, Finland; 2 Turku PET Centre, University of Turku, Turku University HospitalTurku, Finland; 3 Department of Medicine, University of Turku, Turku University HospitalTurku, Finland; 4 Department of Biology of Physical Activity, University of JyväskyläJyväskylä, Finland; 5 Department of Pathology, Haartman Institute, University of HelsinkiHelsinki, Finland; 6 HUSLAB, Division of Pathology, Helsinki University Central HospitalHelsinki, Finland; 7 Department of General Surgery, Leiden University Medical CenterLeiden, The Netherlands; 8 Oulu Center for Cell-Matrix Research, Biocenter Oulu and Department of Medical Biochemistry and Molecular Biology, University of OuluOulu, Finland; 9 Department of Anatomy, University of BernBern, Switzerland; 10 Section of Cardiovascular Medicine, Department of Internal Medicine, Yale University School of MedicineNew Haven, CT, USA; 11 Institute of Biomedicine, Pharmacology, University of HelsinkiHelsinki, Finland

**Keywords:** angiogenesis, endothelial cell, ischemia, metabolism, VEGF-B

## Abstract

Angiogenic growth factors have recently been linked to tissue metabolism. We have used genetic gain- and loss-of function models to elucidate the effects and mechanisms of action of vascular endothelial growth factor-B (VEGF-B) in the heart. A cardiomyocyte-specific VEGF-B transgene induced an expanded coronary arterial tree and reprogramming of cardiomyocyte metabolism. This was associated with protection against myocardial infarction and preservation of mitochondrial complex I function upon ischemia-reperfusion. VEGF-B increased VEGF signals via VEGF receptor-2 to activate Erk1/2, which resulted in vascular growth. Akt and mTORC1 pathways were upregulated and AMPK downregulated, readjusting cardiomyocyte metabolic pathways to favor glucose oxidation and macromolecular biosynthesis. However, contrasting with a previous theory, there was no difference in fatty acid uptake by the heart between the VEGF-B transgenic, gene-targeted or wildtype rats. Importantly, we also show that VEGF-B expression is reduced in human heart disease. Our data indicate that VEGF-B could be used to increase the coronary vasculature and to reprogram myocardial metabolism to improve cardiac function in ischemic heart disease.

**Subject Categories** Cardiovascular System; Metabolism

See also: **C Kupatt and R Hinkel** (March 2014)

## Introduction

Paracrine signaling between the major cell types in the heart (cardiomyocytes, endothelial and smooth muscle cells, and fibroblasts) is important for normal cardiac development and function, as well as for the remodeling and repair of damaged and diseased myocardium (Tirziu *et al*, 2010; Doroudgar ' Glembotski, 2011). In physiological and compensated hypertrophy, there is a coordinated regulation of cardiac growth, metabolism and coronary angiogenesis (Shiojima *et al*, 2005; Sano *et al*, 2007; Tirziu *et al*, 2007), but the responsible signals and mechanisms are not fully understood. Recently, a link between vascular endothelial growth factor (VEGF), VEGF-B and regulation of tissue metabolism has been established (Arany *et al*, 2008; Hagberg *et al*, 2010, 2012). A better understanding of the factors that regulate myocardial angiogenesis and metabolism could lead to the development of new therapies for the treatment of heart failure, which is one of the most common causes of morbidity and mortality in developed countries.

Members of the VEGF family, comprising five mammalian proteins, are major regulators of blood and lymphatic vessel development and growth (Lohela *et al*, 2009). Until recently, VEGF-B has been regarded as an exception in the family, as efforts to promote angiogenesis with VEGF-B have given largely negative results (Rissanen *et al*, 2003; Karpanen *et al*, 2008). VEGF-B is highly expressed in cardiomyocytes; however, mice lacking VEGF-B are viable and display mild cardiac phenotypes, such as a slightly smaller heart size and dysfunctional coronary vasculature in one strain (Bellomo *et al*, 2000) and a prolonged PQ interval in the electrocardiogram in another strain (Aase *et al*, 2001). On the other hand, an overdose of VEGF-B via adenoviral delivery into the myocardium transiently enlarged myocardial vessels (Lahteenvuo *et al*, 2009; Serpi *et al*, 2011), and ameliorated angiotensin II-induced diastolic dysfunction (Serpi *et al*, 2011), while transgenic (TG) overexpression of VEGF-B in the rat myocardium induced cardiac hypertrophy and growth of the epicardial and subendocardial coronary vessels (Bry *et al*, 2010). Furthermore, adeno-associated virus (AAV)-mediated administration of VEGF-B_167_ preserved cardiac contractility in rats after experimental myocardial infarction (Zentilin *et al*, 2010), as well as in dogs subjected to tachypacing-induced dilated cardiomyopathy (Pepe *et al*, 2010). Interestingly, placenta growth factor (PlGF), which binds to the same receptors as VEGF-B, was recently shown to induce myocardial angiogenesis and cardiac hypertrophy through an NO-dependent mechanism via the Akt/mTORC1 pathway (Jaba *et al*, 2013). However, in another report, PlGF only secondarily supported pressure-overload induced cardiac hypertrophy through a paracrine mechanism via endothelial cells and fibroblasts (Accornero *et al*, 2011).

Interestingly, VEGF-B and mitochondrial gene expression are coordinately regulated (Mootha *et al*, 2003; Hagberg *et al*, 2010), and endogenous VEGF-B levels are highest in tissues with high metabolic activity, such as the heart, skeletal muscle and brown adipose tissue (Olofsson *et al*, 1996; Aase *et al*, 1999). The absence of VEGF-B was reported to lead to decreased expression of fatty acid (FA) transport proteins (Fatp3 and Fatp4) in endothelial cells, which correlated with decreased lipid droplets in cardiomyocytes and skeletal muscle fibers (Hagberg *et al*, 2010), and improved insulin sensitivity in diabetic models (Hagberg *et al*, 2012).

We show here that VEGF-B increases functional coronary vasculature, reprograms cardiomyocyte metabolic pathways and protects the rat heart from ischemic damage. In addition, we show that the expression of VEGF-B is decreased in human heart disease. However, in contrast to recent studies using VEGF-B deleted mice, fatty acid uptake was not significantly changed in VEGF-B deficient or VEGF-B overexpressing rats. Overall, our results indicate that VEGF-B has therapeutic potential, as the cardiac hypertrophy induced by VEGF-B does not progress into pathological cardiac remodeling or heart failure even in aged rats, and the VEGF-B induced hypertrophic and metabolic changes are beneficial in myocardial ischemia.

## Results

### VEGF-B TG rats have a significantly enhanced functional coronary vasculature

**Figure 1:**
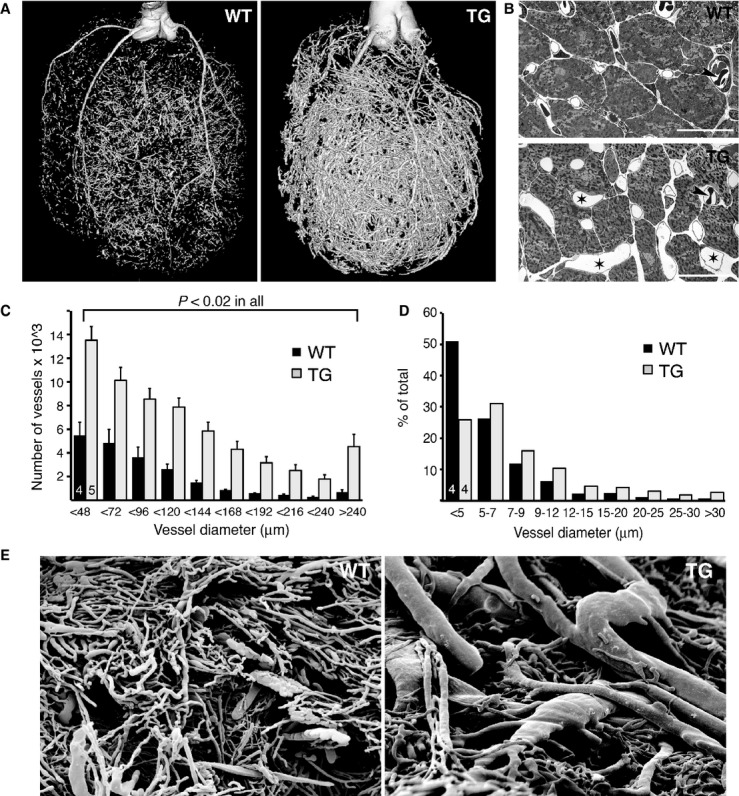
Increased coronary vasculature in aMHC-VEGF-B TG rats.
Representative arterial μCT images of WT and TG hearts at 3 months of age.Transmission electron micrographs (TEM) of WT and TG hearts. TG rats had numerous large capillaries and microvessels (asterisks). Arrowheads indicate erythrocytes of the same size in both groups. Scale bars = 20 μm.Quantification of the number of vessels of various sizes from two-dimensional transverse μCT images.Quantification of the relative percentages of subendocardial capillaries of different sizes from the TEM images. The number of rats used is shown in the columns.Scanning electron microscopic images of vascular casting. Note several large vessels in the subendocardium of the VEGF-B TG hearts. Data information: Data are shown as mean ± s.e.m. (Student's *t*-test). This figure is accompanied by supplementary Fig 1.

To study the effects of VEGF-B on the entire coronary arterial tree, coronary arteries were filled *ex vivo* with a contrast agent and analyzed with high-resolution micro-computed tomography (μCT). This revealed a striking increase in arteries of all sizes in the VEGF-B TG hearts when compared to wildtype (WT) controls (Fig 1A and C). The increase was about two-fold in arteries of <100 μm diameter, whereas in the larger vessels (>150 μm), the increase was more than five-fold. Since the increase in cardiomyocyte size and heart weight was about 20–30% (Bry *et al*, 2010), this indicated an increased arteriole/cardiomyocyte-ratio. In addition, transmission electron microscopy revealed capillaries with increased diameter in the TG hearts (Fig 1B and D), and scanning electron microscopy confirmed the presence of large vessels in the subendocardium (Fig 1E).

### The cardiac hypertrophy is physiological even in old VEGF-B TG rats

The TG rats showed more arterialized vessels in the heart (Supplementary Fig 1A), and an increased heart-to-body weight ratio compared to WT rats already at postnatal day 8 (7.9 ± 0.6 TG versus 4.7 ± 0.1 mg/g WT, *P *=* *0.00001). The increased heart-to-body weight ratio was maintained in 2-month-old as well as in aged 22-month-old rats (*P *=* *0.001) (supplementary Fig 1B). Echocardiography revealed that the ejection fraction and fractional shortening were maintained in the old TG rats, and the stroke volume was increased (supplementary Table 1). Maximal exercise capacity did not differ significantly between the TG and WT rats (471 ± 88 versus 412 ± 83 m, respectively), and there were no differences in maximal oxygen uptake or carbon dioxide production (supplementary Fig 1C and D). The amount of connective tissue was also similar in the WT and TG rats at the respective time points (supplementary Fig 1E).

To further determine the nature of the VEGF-B induced hypertrophy, we analyzed the expression of genes associated with pathological remodeling. There were no differences between the genotypes in *Anp*, *Bnp*, *Myh6*, *Myh7* or *ActA* gene expression, or in the *Myh7/Myh6* ratio (supplementary Fig 1F), confirming that the hypertrophy was physiological rather than pathological. Among exercise-induced transcription factors associated with hypertrophy (Bostrom *et al*, 2010), only the RNA encoding CyclinD1 (*Ccnd1)* was significantly upregulated in the TG rats (*P *=* *0.006) (supplementary Fig 1G).

### VEGF-B TG hearts are protected from ischemic damage

To study whether the increased vasculature seen in the VEGF-B TG rats was functional and could provide protection from ischemic myocardial damage, TG and WT rats were subjected to experimental myocardial infarction (MI). Echocardiography was performed before ligation of the left coronary artery as well as 1 and 4 weeks after the MI. The decrease in the ejection fraction and fractional shortening as well as the increase in left ventricular systolic and diastolic diameters as a result of the MI were markedly less severe in the TG rats when compared to their WT controls both 1 and 4 weeks after the MI (supplementary Fig 2A–C, *P* = 0.0001–0.02).

**Figure 2:**
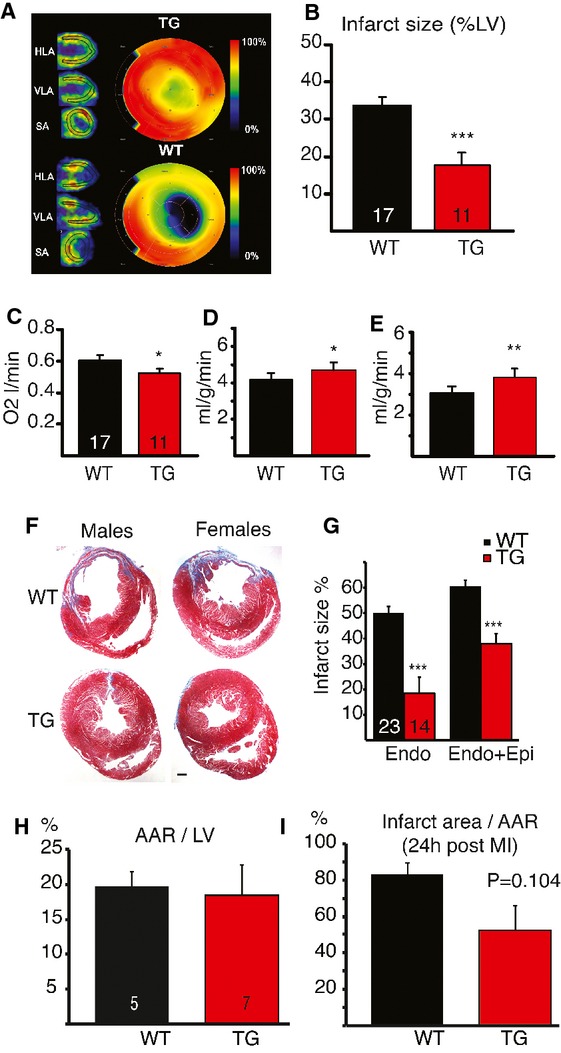
Infarct size and cardiac perfusion in VEGF-B TG and WT rats. A  Representative PET images and polar maps of the left ventricle (LV) myocardial perfusion using 11C-acetate 4 weeks after the MI (***P = 0.0001). B  Quantification of the MI size in 11C-acetate PET perfusion images. C  Oxygen consumption measured by 11C-acetate PET in the non-infarcted myocardium (*P = 0.016) D  Perfusion in non-infarcted myocardium (*P = 0.046) E  Residual perfusion in infarcted and border areas (**P = 0.006). F  Masson's trichrome staining of heart sections 4 weeks after the MI. G  Quantification of scar tissue area in the endocardial side (***P = 6.1 × 10^−6^) or epicardial plus endocardial sides (***P = 7.5 × 10^−6^). H, I  (H) Area-at-risk (AAR) and (I) infarct area in relation to AAR were evaluated by Evans blue perfusion and TTC staining 24 h after MI. The number of rats used is shown in the columns. Data information: Data are shown as mean ± s.e.m. (Student's *t*-test). This figure is accompanied by supplementary Fig 2.

Positron emission tomography (PET) using 11C-acetate perfusion showed a significantly smaller infarct region in the TG hearts 4 weeks after the MI (*P *=* *0.0001, Fig 2A and B), while the non-infarcted TG myocardium consumed less oxygen than the WT myocardium (*P = *0.02) (Fig 2C). Furthermore, the TG hearts showed a better perfusion of the non-infarcted septum (*P *=* *0.04, Fig 2D), and a better residual perfusion of the infarcted and border areas (*P *=* *0.006, Fig 2E).

Post mortem and histological analysis of the hearts confirmed that the infarct and scar tissue areas were smaller in the VEGF-B TG hearts (Fig 2F and G). The remote myocardium in the TG hearts had an increased total arterial area and larger capillaries than the WT hearts (supplementary Fig 2D–H), similarly to what was observed in non-infarcted hearts. The scar tissue in the infarcted TG hearts also had fewer myofibroblasts than in the WT hearts (supplementary Fig 2I and J). In line with the perfusion data, the TG rat hearts also had more arteries in the border areas of the infarction scars (13.2 ± 1.8 versus 8.2 ± 0.8 arteries/field, *P *=* *0.013).

### VEGF-B protects cardiomyocyte mitochondria from ischemia/reperfusion injury

Since the coronary vasculature was enhanced in the VEGF-B TG hearts, we assessed the myocardial area-at-risk (AAR) upon coronary artery ligation as well as the infarct areas 24 h after the MI. The AAR was comparable in the WT and TG hearts, but there was a trend towards smaller scars in TG hearts already 24 h after ligation (Fig 2H and I). Thus, although the initial ischemic insults were comparable in size, the TG cardiomyocytes were better protected from ischemic damage. There were no differences in the inflammatory response in the infarct/border area 24 h or 4 weeks after the MI, but in the remote myocardium expression of CD68 (macrophages) was significantly higher in TG rats 4 weeks after the MI (2.8–fold upregulation, *P *=* *0.0003).

**Figure 3:**
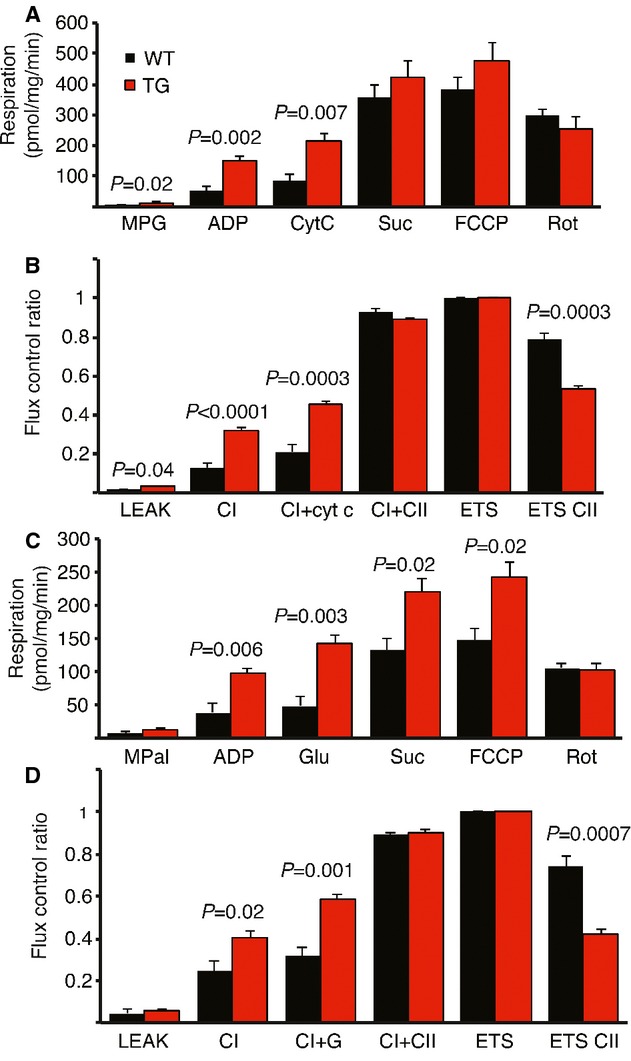
Mitochondrial complex I function is maintained in TG hearts after ischemia/reperfusion.
Mitochondrial respiration with carbohydrate substrates.Flux control ratios normalized for physiological non-coupled respiration (ETS) with carbohydrates.Mitochondrial respiration with palmitoylcarnitine as a substrate.Flux control ratios with palmitoylcarnitine. Data information: Data are shown as mean ± s.e.m. (Student's *t*-test), *N* = 4 WT + 5 TG. (MPG = malate, pyruvate, glutamate; CytC = cytochrome C; Suc = succinate; FCCP = carbonilcyanide p-triflouromethoxyphenylhydrazone; Rot = rotenone; MPal = malate, palmitoylcarnitine; Glu = glutamate; CI/II = complex I/II; ETS = electron transfer system).

To assess the potential protective mechanism of VEGF-B in cardiomyocytes, we performed an ischemia/reperfusion (I/R) experiment, which mimics therapeutic reperfusion in human patients. The hearts were subjected to ischemia by ligating the left anterior descending (LAD) coronary artery for 30 min, followed by reperfusion for 2 h. Mitochondrial function was analyzed from the ischemic areas using carbohydrate and FA substrates. Mitochondrial respiratory chain complex I activity was significantly better maintained in the TG hearts using both carbohydrates and FAs, providing strong evidence that VEGF-B protected cardiomyocyte mitochondria during the I/R stress (Fig. 3). In basal conditions, there were no differences in mitochondrial function between the WT and TG hearts.

### VEGF-B deficient rats have normal vasculature in the heart

In order to study the effects of VEGF-B deficiency in rats, we generated a VEGF-B knockout (KO) rat model. The construct and verification of the model is presented in supplementary Fig 3. There was no difference in the body weight or weight of the heart, lung, liver, kidney, epididymal fat pads or interscapular brown adipose tissue between WT and KO rats at 8 weeks of age. Furthermore, the capillary diameter (6.1 ± 0.1 versus 6.3 ± 0.2 μm), capillary density (1313 ± 34 versus 1224 ± 35 capillaries/field) and average arterial size (87 ± 6 versus 85 ± 7 μm^2^) and number (5.7 ± 0.6 versus 6.2 ± 1.0 arteries/field) were similar between the WT and KO hearts. The KO rats and controls were also subjected to MI, where no differences in cardiac function could be seen during a 16-week follow-up. Interestingly however, in histochemical analyses 16 weeks post-MI, the two-dimensional infarct size was significantly larger in KO compared to WT rats (43.4 ± 3.6% of endocardial length in KO versus 23.8 ± 4.8% in WT, *P *=* *0.004 and 58.7 ± 3.5% of epi- and endocardial length in KO versus 36.3 ± 3.6% in WT, *P *=* *0.0005), suggesting mild dysfunction of the vasculature in KO rat hearts in this pathological setting.

### AAV-VEGF-B administration mimics the effects of the VEGF-B transgene

**Figure 4:**
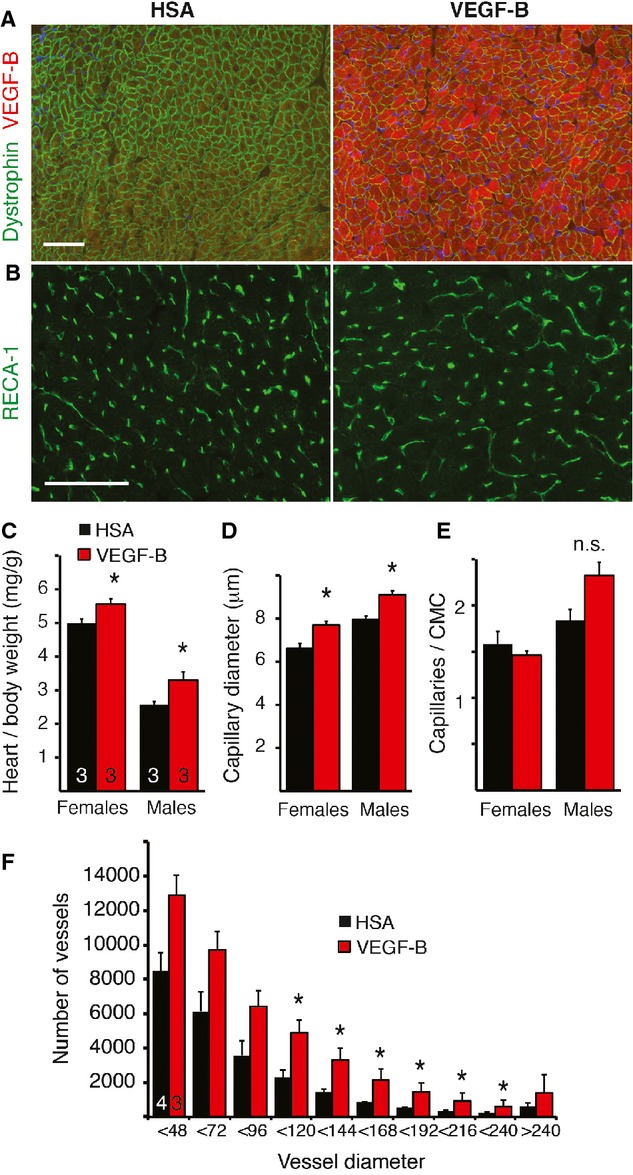
AAV-VEGF-B induces cardiac hypertrophy and increased capillary size and number of arteries in adult rats. A Immunofluorescence staining of VEGF-B in the myocardium 2 months after systemic administration of AAV-VEGF-B. B Representative images of RECA-1 staining for endothelial cells. C Quantification of heart-to-body weight ratios two (females, *P = 0.046) and four (males, *P = 0.043) months after the treatment. D, E Quantification of the capillary size (females *P = 0.0016, males *P = 0.033) and density (CMC, cardiomyocyte; n.s., not significant; *P* = 0.06). F Quantification of μCT analysis of the arterial tree in AAV-VEGF-B treated and control hearts, *P < 0.05. Data information: Data are shown as mean ± s.e.m. (Student's *t*-test). Scale bars = 100 μm.

To address the therapeutic potential of VEGF-B, we analyzed if the phenotype of the VEGF-B TG hearts is reproducible via AAV-gene transfer to adult rats. We confirmed VEGF-B expression in the heart by immunofluorescence staining and western blotting after AAV-VEGF-B administration via a tail vein (Fig 4A). A significantly increased heart-to-body weight ratio was observed when the vector was expressed for 2 months in female rats or 4 months in male rats (Fig 4C). As in the TG hearts, the AAV-VEGF-B transduced hearts also had larger capillaries and a maintained capillary-to-cardiomyocyte ratio (Fig 4B, D and E). Importantly, μCT imaging confirmed an approximately 2.5-fold increase in the number of coronary arteries of 100–240 μm in diameter in AAV-VEGF-B hearts compared to AAV-human serum albumin (HSA) hearts (Fig 4F).

Quantification of leukocytes by CD45-immunostaining (21.6 ± 5.2 cells/field in AAV-VEGF-B versus 19.0 ± 4.4 cells/field in AAV-HSA hearts, *P = *0.84) or ED-1 -positive macrophages (*P = *0.86) did not reveal increased inflammatory cells in AAV-VEGF-B hearts. Furthermore, AAV-VEGF-B and AAV-HSA injected rats showed no differences in a spectrum of clinical serum parameters including blood electrolytes, glucose, lipoproteins or liver enzymes.

### Upregulation of blood vasculature development pathways in VEGF-B overexpressing hearts

**Figure 5:**
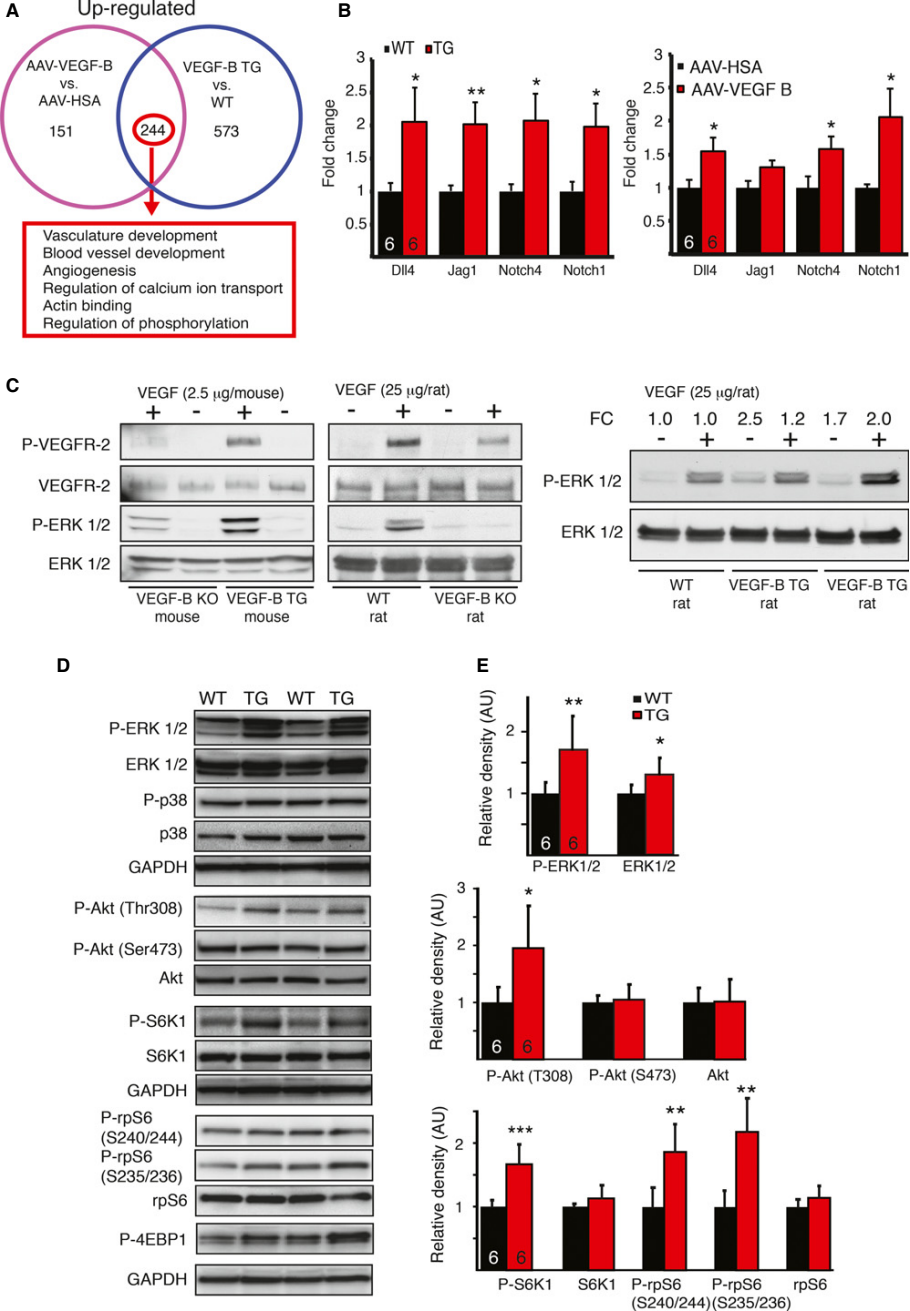
VEGF-B induces angiogenic gene expression and Erk1/2/Akt/mTORC1 signaling by potentiating the effects of VEGF.
Venn-diagram and functional clustering of genes upregulated by VEGF-B (*N* = 6 + 6 in both experiments).mRNA expression of genes involved in the Notch signaling pathway. TG versus WT Dll4 *P = 0.046, Jag1 **P = 0.009, Notch4 *P = 0.017, Notch1 *P = 0.016; AAV-VEGF-B versus AAV-HSA Dll4 *P = 0.033, Jag1 *P* = 0.14, Notch4 *P = 0.031, Notch1 *P = 0.037.*In vivo* -signal transduction 10 min after i.v. injection of VEGF to VEGF-B KO and TG mouse hearts and to VEGF-B KO, TG and WT rat hearts. VEGF induced phosphorylation of VEGFR-2 and Erk1/2; however the effect was stronger in VEGF-B TG mice than VEGF-B KO mice and in WT rat than in KO rats. A stronger effect was also seen in TG rats compared to WT rats. FC = fold-change.Representative immunoblots of cardiac protein extracts probed with antibodies against phosphorylated and total Erk1/2, p38, Akt, S6K1, rpS6 and P-4EBP1.Densitometric quantification of the blots. All signals were normalized to total protein (AU: arbitrary units) and the exact p-values are reported in the text. *P < 0.05, **P < 0.01, ***P < 0.001. Data information: Data are shown as mean ± s.e.m. The number of hearts analyzed is indicated in the bars (Student's t-test). This figure is accompanied by supplementary Figs 3 and 4.

Genome-wide RNA microarray analysis identified 244 significantly upregulated and 40 downregulated genes, which were found in both VEGF-B TG and AAV-VEGF-B hearts (supplementary Table 2). Functional clustering revealed that the most significantly upregulated gene ontologies were related to blood vessel growth and angiogenesis, which supports the observed vascular phenotype of the rats (Fig 5A and supplementary Table 3). Furthermore, the Notch signaling pathway and transcripts involved in the regulation of calcium transport and actin binding were upregulated in the TG and AAV-VEGF-B hearts (Fig 5B).

### VEGF-B increases VEGF–VEGFR-2 signal transduction in the heart

In order to explore the mechanistic basis for the VEGF-B signals, we stimulated VEGF-B KO and TG mouse hearts with VEGF_165_ protein via tail vein injection. We detected increased VEGFR-2 and Erk1/2 phosphorylation in the VEGF-B TG hearts when compared to the VEGF-B KO hearts after 10 min of VEGF stimulation (Fig 5C and supplementary Fig 4A), suggesting that an excess of VEGF-B in the heart allowed more VEGF to bind to and activate VEGFR-2. We furthermore confirmed that VEGF stimulated more Erk1/2 phosphorylation in the TG compared to WT and in WT compared to KO rat hearts (Fig 5C).

### VEGF-B induces activation of Erk1/2, Akt, and mTORC1 pathways

The downstream signaling of VEGF-B has not previously been studied in detail. Protein homogenates from the left ventricle of the TG rat hearts were analyzed for major growth factor signal transduction pathways (Fig 5D and E). Overexpression of VEGF-B induced strong Erk1/2 (Thr^202^/Tyr^204^) phosphorylation (*P *=* *0.0097), whereas no change was observed in p38 Thr^180^/Tyr^182^ or Akt Ser^473^ phosphorylation. However, phosphorylation of Akt at Thr^308^ (*P *=* *0.02) and of the downstream components of the mTORC1 pathway S6K1 (Thr^389^) (*P *=* *0.0004) and rpS6 (Ser^240/244^ and Ser^235/236^) (*P *=* *0.002 and 0.001) were significantly increased in the VEGF-B TG hearts. Phosphorylation of Akt at Ser^473^ and at Thr^308^ is considered to reflect the activation of mTORC2 and mTORC1, respectively (Shiojima ' Walsh, 2006). There was also a trend towards increased 4EBP1 phosphorylation (*P *=* *0.06) in the TG hearts. Immunohistochemistry further showed strong phosphorylation of rpS6 in arterial smooth muscle cells in the TG hearts, and a somewhat increased signal in the capillary endothelium and in the cardiomyocytes (supplementary Fig 4C). These results indicate that VEGF-B signaling engages major regulators of cell growth and metabolism.

### Soluble VEGFR-2 inhibits the VEGF-B-induced capillary enlargement

**Figure 6:**
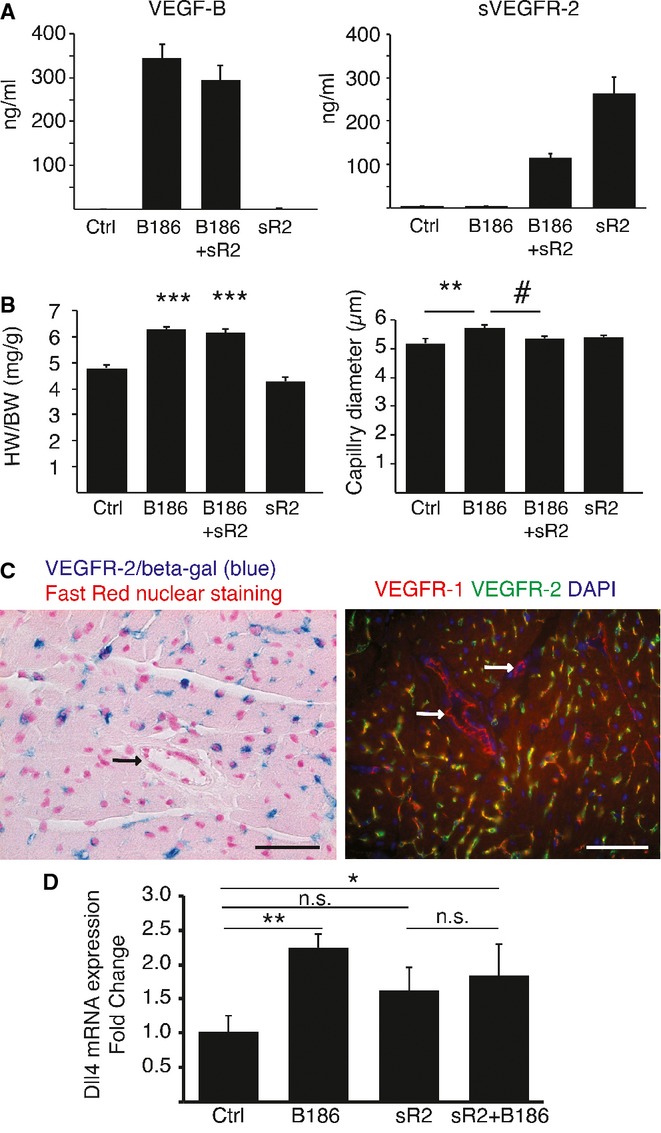
Blocking VEGF-VEGFR2 signaling inhibits VEGF-B induced capillary enlargement.
Serum levels of VEGF-B186 (B186) and soluble VEGFR-2 (sR2) 4 weeks after AAV injections.Effect of soluble VEGFR-2 and VEGF-B alone or in combination. Note that sR2 does not seem to change the VEGF-B induced heart-to-body weight –ratio but blocks the increase in capillary diameter induced by VEGF-B. ***P < 0.0001, **P = 0.002 compared to controls ^#^*P* = 0.014 compared to VEGF-B186.Beta-galactosidase staining of the VEGFR-2/LacZ mouse heart. Note VEGFR-2 expression in capillary endothelium but not in arteries (arrow) or cardiomyocytes. Double staining for VEGFR-1 (red) and VEGFR-2 (green) in mouse heart. Note expression of both in the capillary endothelium but only VEGFR-1 in the arterial endothelium (arrows). Scale bars 50 μm.Dll4 mRNA expression, used as an arterial marker in the heart. **P = 0.003, *P = 0.02, n.s. = non-significant. Data information: Data are shown as mean ± s.e.m. (one-way ANOVA with LSD post hoc test).

We have previously shown that VEGFR-1 tyrosine kinase activity plays a role in the VEGF-B–induced myocardial hypertrophy (Bry *et al*, 2010). To investigate the role of VEGFR-2, we conducted a VEGF-blocking experiment using soluble (s) VEGFR-2 coupled with VEGF-B overexpression in mice. Administration of an AAV encoding sVEGFR-2 did not significantly affect the hypertrophy induced by AAV-VEGF-B, but it blocked the enlargement of capillaries (Fig 6A and B). Interestingly, by immunostaining and staining for beta-galactosidase activity in heterozygous VEGFR-2/LacZ mice, we found that both VEGFR-1 and VEGFR-2 are present in myocardial capillaries whereas only VEGFR-1 is present in the coronary arteries (Fig 6C). AAV-mediated administration of mouse VEGF-B_186_ increased expression of the arterial marker Dll4 when combined with an empty vector, but not when co-injected with AAV-sVEGFR-2, indicating that sVEGFR-2 affected also the VEGF-B induced arteriogenic response (Fig 6D).

### VEGF-B-induced hypertrophy is not dependent on nitric oxide signaling

A recent publication indicates that PlGF induces cardiac hypertrophy via a nitric oxide (NO) mediated mechanism (Jaba *et al*, 2013). To test if this mechanism applies to the VEGF-B induced cardiac growth, we administered AAV-mVEGF-B186 to endothelial nitric oxide synthase (eNOS) deficient mice as well as to mice treated with the NOS inhibitor L-NAME. Interestingly, blocking NO production did not have any effect on VEGF-B-induced cardiac hypertrophy (supplementary Fig 5).

### VEGF-B overexpressing hearts shift from fatty acid to glucose oxidation pathways

**Figure 7:**
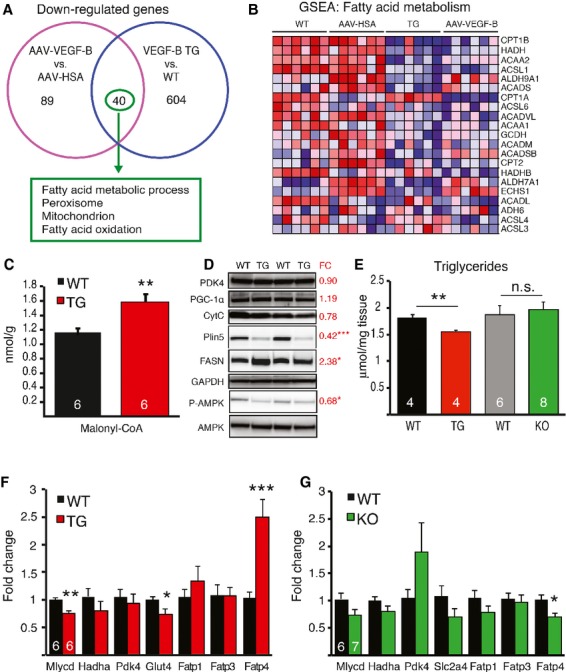
VEGF-B induced metabolic changes in the heart.
Venn-diagram and functional clustering of genes downregulated by VEGF-B (*N* = 6 + 6 in both experiments). Functional clustering showed that most of the forty genes downregulated in both TG and AAV-VEGF-B hearts are related to fatty acid metabolism.Heat map from the GSEA analysis, showing significant downregulation of genes related to fatty acid degradation in both AAV-VEGF-B and VEGF-B TG hearts.Malonyl-CoA content was significantly increased in TG hearts. *P = 0.005AMPK phosphorylation (*P = 0.019) and perilipin 5 expression (***P = 3.8 × 10^−5^) were significantly reduced in TG hearts, whereas the expression of fatty acid synthase (FASN) was increased (*P = 0.022). There was no change in pyruvate dehydrogenase kinase 4 (PDK4), PGC-1α or cytochrome c (CytC). Fold change (FC) shows the relative expression in the TG hearts compared to the WT hearts (*N* = 6 + 6).Triglyceride content was reduced in the TG hearts (**P = 0.007) but not changed in the KO hearts. Expression of metabolic genes in TG (F) and KO (G) hearts compared to their respective controls. Data information: Data are shown as mean ± s.e.m. (Student's *t*-test). This figure is accompanied by supplementary Fig 6.

To our surprise, the most significantly downregulated gene cluster in the VEGF-B transgenic hearts comprised genes that function in fatty acid metabolism (supplementary Table 4). Gene Set Enrichment Analysis (GSEA) of unfiltered data from both the TG and AAV sample sets confirmed this result (Fig 7A and B). In contrast, most of the intermediate products of glycogen breakdown and glycolysis were increased in the VEGF-B TG hearts when compared to WT hearts (supplementary Fig 6). To further analyze the mechanisms of the metabolic adaptation in the VEGF-B TG hearts, we studied the expression of several key genes and proteins related to metabolic regulation. Phosphorylation of AMPK was significantly reduced in the TG hearts and in WT hearts after acute VEGF and VEGF-B stimulation (Fig 7D and supplementary Fig 4B). There was also a trend towards decreased acetyl-CoA carboxylase phosphorylation. This was accompanied with increased malonyl-CoA levels and decreased malonyl-CoA decarboxylase (*Mlycd*) RNA expression together with increased fatty acid synthase (FASN) protein expression in the TG hearts (Fig 7C, D and F). These changes indicated that decreased fatty acid oxidation and increased lipid/macromolecule synthesis support the cell growth associated with the cardiac hypertrophy and vessel growth.

### Fatty acid uptake is not altered in VEGF-B TG or KO hearts

Despite the decreased expression of beta-oxidation genes, the levels of long-chain free FAs and triglycerides were lower in the TG than in the WT hearts (Fig 7E and supplementary Table 5). This was supported by the finding that one of the most downregulated RNAs in the microarray analysis of the TG hearts was perilipin 5. This major lipid droplet coating protein was decreased also in western blotting analysis (Fig. 7D). There were no differences in total tissue cholesterol (4.0 ± 0.1 μmol/mg in WT versus 4.1 ± 0.2 in TG), phospholipid levels (10.4 ± 0.5 μmol/mg in WT versus 10.3 ± 0.9 in TG), or serum free fatty acid and triglyceride levels. Co-expression of *Vegfb* RNA and various RNAs encoding mitochondrial proteins has been reported in large data sets (Mootha *et al*, 2003; Hagberg *et al*, 2010). However, VEGF-B did not seem to affect mitochondrial biogenesis, as PGC-1α and cytochrome c protein levels were similar in the WT and TG rat hearts (Fig 7D). In agreement with previous reports, we observed decreased fatty acid transport protein 4 (*Fatp4/Slc27a4*) mRNA levels in the KO hearts and increased levels in the TG hearts (Hagberg *et al*, 2010), but no change in *Fatp1* or *Fatp3* expression in either model (Fig 7F, G).

**Figure 8:**
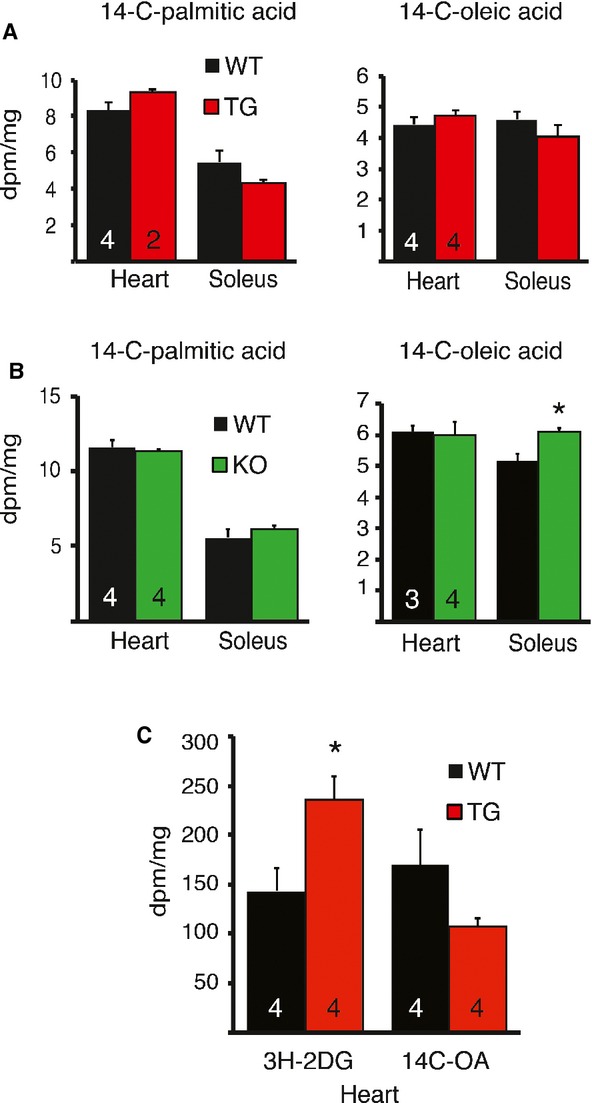
No difference in fatty acid uptake in the VEGF-B TG or KO rat hearts.
14C-oleate and 14C-palmitate uptake in the heart and skeletal (soleus) muscle of TG orKO rats 24 h after oral gavage. Note the slightly increased uptake in KO soleus muscle, *P = 0.011.Glucose uptake and oleate uptake 30 min after tail vein injection of substrates. *P = 0.035. Data information: Data are shown as mean ± s.e.m. (Student's *t*-test).

Since loss of VEGF-B has been reported to decrease the uptake of FAs in the mouse heart and skeletal muscle (Hagberg *et al*, 2010), we tested if FA uptake was affected in the TG and KO rat models. We did not observe a decrease in ^14^C-oleate or ^14^C-palmitate uptake in the KO rats nor an increase in the TG rats 24 h after ingestion of the radioactive FAs (Fig 8A, B). Using tail vein injection of labeled glucose and FA, we found increased glucose uptake in the TG hearts compared to the WT hearts 30 min after injection, whereas there was no significant difference in FA uptake (Fig 8C).

### Vascular remodeling precedes the metabolic changes

In order to see if the metabolic changes in the VEGF-B transduced hearts are mediated directly or represent adaptation to long-term transgene expression, we overexpressed VEGF-B for 2 weeks in rats using the AAV-vector. Analysis at this time point showed enlarged myocardial capillaries (48% increase, *P *=* *0.007) and microarray analysis indicated increased expression of angiogenic genes similarly as in the longer-term experiments. At this early timepoint, the heart-to-body weight-ratio was not yet significantly changed, but there was a 10% increase in the cross-sectional area of cardiomyocytes in the AAV-VEGF-B hearts (*P *=* *0.01). However, no change was observed in the metabolic gene expression patterns, including *Fatp3* and *Fatp4*. We also analyzed the FA and glucose uptake 2 weeks after AAV-VEGF-B administration. There was no significant difference in the uptake of either substrate between the AAV-VEGF-B and control hearts (214 ± 25 dpm/mg 3H-2DG in WT versus 151 ± 37 in TG, *P *=* *0.23 and 124 ± 5 dpm/mg 14C-OA in WT versus 76 ± 43 in TG, *P *=* *0.34).

### Myocardial VEGF-B expression is decreased in heart failure

**Figure 9:**
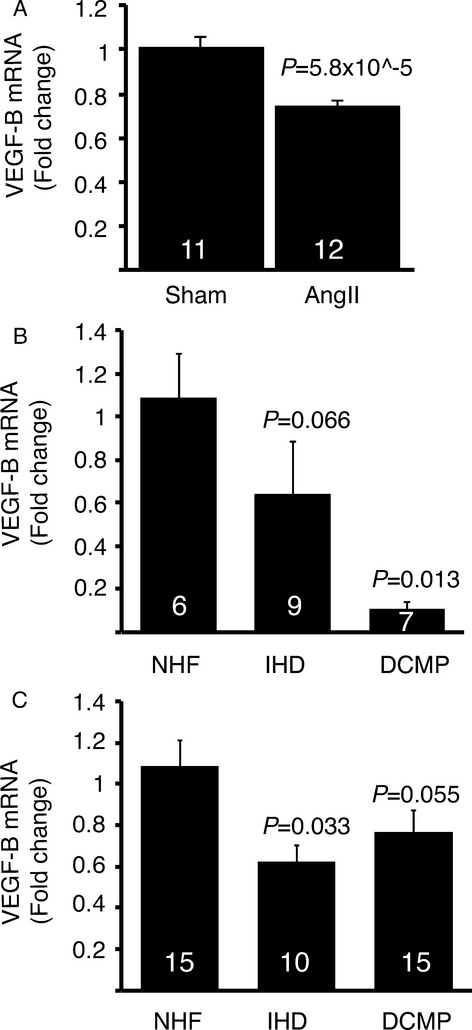
VEGF-B expression is reduced in diseased human myocardium and in angiotensin II treated mouse hearts. A Cardiac VEGF-B expression in mice treated for 2 weeks with angiotensin II compared to control mice (Student's *t*-test). B, C VEGF-B expression in ischemic heart disease (IHD) and dilated cardiomyopathy (DCMP) compared to non-failing hearts in two independent datasets (B: Leiden C: Helsinki). Data information: Data are shown as mean ± s.e.m. (one-way ANOVA with Dunnett's post hoc test). The number of patients/mice in each group is indicated in the bars.

Decreased expression of VEGF-B was observed in mice treated for 2 weeks with angiotensin II (Fig 9A), which provides a model of pathological cardiac hypertrophy. To further explore the translational potential of our findings, we analyzed VEGF-B expression in diseased human myocardium. The results from two independent data sets indicated that VEGF-B expression is significantly decreased in both ischemic heart disease and dilated cardiomyopathy compared to non-failing hearts (Fig 9B and C).

## Discussion

In this study, we show that VEGF-B dramatically expands the coronary arterial tree and increases functional coronary reserve, accompanied by cardiac hypertrophy and increased glucose uptake. Together, these changes provided significant cardiac protection against ischemic damage. Importantly, the cardiac function in VEGF-B TG rats was maintained even in old age, and the hypertrophy did not advance to heart failure.

The increase in functional coronary arterial reserve observed in μCT imaging of the VEGF-B TG hearts is striking, revealing at least a doubling of the number of arteries of all size classes. At the capillary level, the vessel diameter was increased, but the capillary-to-cardiomyocyte ratio was not affected. It is interesting that the effect was strongest in the larger arteries, which are mainly affected in coronary artery disease, whereas PlGF was recently reported to primarily increase capillary density and arteriolar branching, along with cardiac hypertrophy (Jaba *et al*, 2013). Importantly, in contrast to the hypertrophy induced by PlGF, blocking NO signaling did not affect the VEGF-B induced cardiac hypertrophy.

Systemic delivery of the AAV-VEGF-B vector to adult rats resulted in strong VEGF-B expression in the heart, and reproduced both the vascular and hypertrophic phenotypes. These results, together with the observation that the long-term expression does not have adverse effects on cardiac function, are important for the possible future use of VEGF-B as a therapeutic agent in ischemic cardiovascular diseases. Angiogenesis has been shown to be crucial for physiological (adaptive) hypertrophy, as disruption of coordinated tissue growth and angiogenesis leads to heart failure (Shiojima *et al*, 2005; Sano *et al*, 2007). The fact that even the aged VEGF-B TG rats showed preserved cardiac function in spite of the long-term hypertrophy indicates coordination of the vascular and cardiomyocyte growth, emphasizing the importance of endothelial cell – cardiomyocyte crosstalk. The outcome was different from that observed in TG mice, which develop cardiac hypertrophy but lack an arteriogenic phenotype, eventually developing cardiomyopathy (Karpanen *et al*, 2008). In the mice the transgene encoded only the isoform VEGF-B167, whereas in the rats the whole genomic construct encoding both 167 and 186 isoforms was used. The biggest difference we have observed between mice and rats is in the VEGF-B–induced arteriogenesis, which may reflect species and/or strain specific differences in collateral artery formation. Indeed, enlargement of myocardial capillaries was seen in both species and already after 2 weeks of VEGF-B expression in the rats. It is conceivable that the expansion of the arterial tree in the TG rats results from increased pulsatile fluid shear stress initially caused by distal capillary enlargement (reviewed in Schaper, 2009).

Following the MI, the infarct areas were significantly smaller in the VEGF-B TG than in the WT rat hearts although the areas-at-risk after ligation were similar. Also, despite the fact that blood perfusion in the VEGF-B TG and WT hearts was similar in the basal state (Bry *et al*, 2010), the VEGF-B TG hearts had better perfusion in the non-infarcted myocardium and border region after MI, indicating that the significance of the improved coronary collateral reserve and associated changes in substrate utilization becomes apparent in pathological settings. Mechanistically, at least part of this protection was shown to arise from the maintained function of mitochondrial respiratory chain complex I in the TG hearts after ischemia/reperfusion injury. It should be mentioned here that defects in complex I function have been linked to the development of heart failure in both animal and human studies (Lemieux *et al*, 2011; Karamanlidis *et al*, 2013).

Some previous studies have also suggested a protective role for VEGF-B in the heart (Lahteenvuo *et al*, 2009; Serpi *et al*, 2011). However, the adenoviral-mediated expression of VEGF-B used in these studies is robust and transient, and its effects were modest compared to our present findings after MI. Zentilin *et al* (2010) reported that AAV-VEGF-B_167_ has direct antiapoptotic effects on cardiomyocytes following experimental myocardial infarction in rats but no obvious vascular phenotype. The various VEGF-B studies thus suggest distinct but complementary roles for VEGF-B in the maintenance of cardiac contractility and coronary perfusion, and our present study indicates mechanisms involving enhanced coronary vasculature and metabolic reprogramming.

We did not observe any vascular phenotype in the hearts of the VEGF-B KO rats. In addition, VEGF-B deficiency did not affect cardiac function, even after MI. However, the infarct scars were two-dimensionally larger in the KO hearts, which might reflect coronary artery dysfunction similarly to that previously reported for VEGF-B KO mice (Bellomo *et al*, 2000). These data point to compensatory mechanisms for the maintenance of the vasculature both during development as well as postnatally, at least with constitutive gene-deletion.

Several major signaling pathways were activated downstream of VEGF-B in the heart, including the Akt/mTORC1 and Erk1/2 MAPK pathways, known to be associated with *e.g*. cardiomyocyte growth and arteriogenesis (Ren *et al*, 2010; Rose *et al*, 2010; Sussman *et al*, 2011). Downstream of Erk1/2 and Akt (T308), VEGF-B activated the mTORC1 complex, which is an important metabolic node involved in the development of cardiac hypertrophy (Shiojima ' Walsh, 2006). The mTORC1 pathway has been related to the regulation of protein synthesis, cardiac function, myocardial response to stress, and myocyte survival (Zhang *et al*, 2010), and it has been shown to be activated by physiological hypertrophy and inactivated by pressure overload (Kemi *et al*, 2008). Interestingly, VEGF-B also decreased AMPK phosphorylation. Recently in another study, increased MAPK and decreased AMPK signaling were related to protection from ischemia-reperfusion injury (McLean *et al*, 2013). However, activation of AMPK has been shown to play an important role in the myocardial response to ischemia, pressure overload, and heart failure (recently reviewed by Zaha ' Young, 2012).

Decreased phosphorylation of VEGFR-2 and Erk1/2 was observed upon intravenous administration of VEGF to VEGF-B KO rats and mice when compared to the corresponding TG or WT animals. This suggests that at least some of the effects of VEGF-B are mediated indirectly by VEGF-B occupying VEGFR-1, which would increase the availability of VEGF for activation of VEGFR-2. This mechanism of vessel growth would be limited by the availability of VEGF; thus the phenotype of VEGF-B overexpression does not recapitulate VEGF overexpression, which can lead to pathological angiogenesis at supraphysiological VEGF doses (Nagy *et al*, 2008; Bry *et al*, 2010; Chung ' Ferrara, 2011). This finding is in agreement with a recent report showing that global deletion of *Vegfr1* in adult mice supports angiogenesis after myocardial infarction by increasing VEGFR-2 levels (Ho *et al*, 2012). The tyrosine kinase domain of VEGFR-1 seems to be required for the VEGF-B induced hypertrophy while neuropilin-1 binding may not be needed (Bry *et al*, 2010). Thus, our results suggest that VEGF-B acts both directly via VEGFR-1 and indirectly via VEGF-VEGFR-2 signaling pathways. In fact, our recent studies have shown that although VEGF-B can bind to VEGFR-1 with high affinity, it cannot induce signaling downstream of VEGFR-1 as efficiently as PlGF (Anisimov *et al*, 2013). VEGF-B, by binding to VEGFR-1, could perhaps also prime VEGFR-2 for enhanced signaling, as has been suggested for PlGF (Autiero *et al*, 2003).

In addition to angiogenesis, cardiac energy substrate metabolism plays a key role in the pathogenesis of heart failure. Collectively, metabolic data indicated increased FA synthesis and decreased FA oxidation in the VEGF-B overexpressing hearts, whereas intermediates of glycogen and glucose breakdown were increased. However, lactate levels and lactate dehydrogenase RNA expression did not change, most likely due to an efficient coupling of the aerobic TCA cycle to the increased glycolysis. Decreased expression of FA oxidation genes, accompanied by a decrease in FA oxidation and a parallel increase in glucose oxidation, are associated with a significant improvement in the recovery of cardiac function after ischemia (Burkart *et al*, 2007), suggesting that shifting energy substrate utilization toward glucose oxidation can improve cardiac function and slow the progression of heart failure (Lopaschuk *et al*, 2010). The VEGF-B–induced metabolic changes may protect cardiomyocyte mitochondria from I/R injury, which occurs during reperfusion, when oxidative metabolism is again activated.

We also analyzed cardiac FA uptake, since Hagberg *et al* (2010) have reported that VEGF-B upregulates endothelial fatty acid transport via Fatp3 and Fatp4. Consistent with their findings, we observed increased *Fatp4* RNA in the TG hearts and decreased levels in the KO hearts. However, there was no difference in oleate or palmitate uptake between TG, KO or WT hearts. It is important to note that fatty acid and triglyceride levels were actually reduced in the TG hearts. Recent evidence suggests that Fatp4 is in fact a fatty acyl-CoA synthase that resides in the endoplasmic reticulum, rather than a fatty acid transporter on the plasma membrane (Digel *et al*, 2011; Lenz *et al*, 2011). Thus while our results confirm that VEGF-B has metabolic effects in the heart, these do not seem to occur at the level of substrate uptake. Instead, VEGF-B readjusts the main metabolic signaling pathways via AMPK and mTORC1 and directs FAs to synthetic pathways rather than to FA oxidation.

It was recently shown that plasma levels of VEGF-B increase after MI in human patients and correlate with preservation of cardiac function, whereas low levels of VEGF-B accurately predict adverse left ventricular remodeling (Devaux *et al*, 2012). We show here that the expression of VEGF-B is significantly decreased in diseased human myocardium, suggesting that enhancing the levels of VEGF-B might also be beneficial in human cardiac patients. The same was true for mice that we treated with angiotensin II and in mice following transverse aortic constriction (Huusko *et al*, 2012), further supporting the translational potential of our current findings.

Proper communication between different cell types in the heart is required to maintain cardiac homeostasis and to build up appropriate responses to stress. It has been suggested that stimulating the expression of endogenous repair-promoting cardiac proteins could be an effective strategy for the prevention of cardiac damage and enhancement of tissue repair (Doroudgar ' Glembotski, 2011). The present data provide evidence for the therapeutic potential of VEGF-B as a protective and repair-enhancing protein in ischemic heart failure, as its overexpression induces favorable changes in the coronary vasculature, cardiac function and myocardial metabolism. Importantly, the therapeutic window for the use of VEGF-B is much wider than for VEGF or VEGF-C, since even high amounts of VEGF-B were well tolerated. In general, our findings highlight the importance of endothelial-cardiomyocyte crosstalk in fine-tuning cardiac responses to stress.

## Materials and Methods

More detailed methods are described in the online Supporting Information.

### VEGF-B TG and KO rats

αMHC-VEGF-B TG rats of outbred HsdBrl:WH Wistar background have been previously described (Bry *et al*, 2010). VEGF-B deficient rats of Sprague-Dawley background were generated with a zinc-finger nuclease based technique by Sigma Advanced Genetic Engineering Labs, Sigma-Aldrich Biotechnology (St. Louis, Missouri, USA). All experiments involving animals were approved by the Provincial State Office of Southern Finland and carried out in accordance with institutional guidelines. TG and WT rats used for the analyses were 2–3 months or 20–22 months old, and in the myocardial infarction experiment 6–7 weeks old.

### MicroCT imaging of the cardiac vessels

High-resolution micro-computed tomography (μCT) imaging was performed in 2-month-old rats as previously published (Tirziu *et al*, 2005). The aorta was cannulated retrogradely proximal to the brachiocephalic trunk. The hearts were perfused with heparin (100 IU/kg) in 0.9% saline followed by adenosine (1 mg/ml). The hearts were then perfusion-fixed with 4% paraformaldehyde, and the coronary arterial tree was filled with contrast agent consisting of 20% bismuth oxychloride (Sigma-Aldrich) in 5% gelatin, until the agent reached the apex, aiming at filling only the arterial vessels. Filled hearts were imaged with a high-resolution μCT imaging system (GE eXplore Locus SP) followed by morphometric analysis of the arterial vessels.

### Immunohistochemistry and immunofluorescence

Paraffin sections were stained with hematoxylin-eosin and Masson's trichrome. The primary antibodies used for immunostaining are described in the Online Data Supplement. Image analysis was carried out using the ImageJ software (NIH).

### Western blotting

The antibodies used are detailed in the Online Supporting Information.

### Experimental myocardial infarction (MI)

Myocardial infarction was induced *in vivo* by ligation of the left coronary artery (LCA). Echocardiography was performed before the operation, as well as 1 and 4 weeks after the operation. The area-at-risk and infarct size were analyzed from another set of animals 24 h after ligation with Evans blue and triphenyltetrazolium chloride (TTC) staining as published previously, with the modification that Evans blue was used instead of phthalocyanine blue (Bohl *et al*, 2009).

### Assessment of myocardial perfusion, infarct size and oxygen consumption with positron emission tomography (PET)

The infarcted rats (11 TG, 17 WT) were imaged with a small animal PET scanner (Inveon or DPET, Siemens, Knoxville, TN, USA) 4 weeks after coronary occlusion. 45 ± 11 MBq of 11C-acetate was administered via the rat tail vein in 0.4–1.0 ml over 10 s. In order to validate measurement of myocardial infarct size by 11C-acetate, a subgroup of the rats (*N *=* *16) was injected with 40 ± 5 MBq of 18F-FDG, a marker of myocardial glucose metabolism and viability in a separate imaging session.

### Mitochondrial function after ischemia-reperfusion

In 5 WT and 5 TG rats, the proximal left anterior descending (LAD) artery was ligated for 30 min and the heart was then reperfused for 2 h. After the reperfusion, samples from the infarcted area were homogenized and immediately analyzed with Oroboros Oxygraph-2k for mitochondrial function. Both carbohydrate and fatty acid (palmitate) SUIT protocols were used as previously described with slight modifications in the injection order (Lemieux *et al*, 2011).

### *In vivo* stimulation with VEGF

VEGF-B deficient mice of C57Bl/6 background (Bellomo *et al*, 2000) and TG mice (Bry *et al*, 2010) backcrossed for eight generations to C57Bl/6 background were injected with 2.5 μg of purified VEGF_165_ protein via a tail vein. Ten minutes after injection, the mice were sacrificed and the left ventricle was prepared and snap-frozen in liquid nitrogen before homogenization for western blotting. A similar experiment was conducted with TG, KO and WT rats with 25 μg of VEGF.

### Microarray analysis

RNA samples from TG versus WT rats and AAV-VEGF-B versus AAV-HSA rats (*N *=* *6 in all groups) were analyzed with the genome-wide Illumina RatRef-12 Expression BeadChip (BD-27-303; Illumina Inc.). Detailed data analyses were performed with the Chipster software as detailed in the supplemental methods (http://chipster.csc.fi) (Kallio *et al*, 2011). The gene array data have been deposited in the Gene Expression Omnibus, accession number GSE38457.

### Fatty acid and glucose uptake *in vivo*

Oleate and palmitate uptake *in vivo* was measured as previously described (Hagberg *et al*, 2010). Briefly, adult rats were administered ^14^C-oleate or ^14^C-palmitate dissolved in olive oil by oral gavage and tissues were collected after 24 h. In another experiment, ^3^H-2-deoxy-glucose or ^14^C-oleate was administered via a tail vein, and hearts and blood were collected 30 min later. Tissues were lysed and radioactivity was measured from serum and lysates by liquid scintillation using Optiphase HiSafe 3 (Perkin-Elmer) and Wallac LS Counter (Turku, Finland).

### Human myocardial samples

#### Leiden dataset

Myocardial tissue samples were taken from patients undergoing cardiac surgery. The patient population has been described previously (Kortekaas *et al*, 2013). Baseline samples were taken before the surgery from dilated cardiomyopathy (*n* = 7), ischemic cardiomyopathy (*n* = 9) or from non-heart failure (*n* = 6) patients, and they were analyzed in the present study. This study was carried out in accordance with the Declaration of Helsinki and was approved by the local ethics committee. All patients provided written informed consent.

#### Helsinki dataset

Human myocardial samples were obtained from patients undergoing cardiac transplantation due to dilated cardiomyopathy (*n* = 15) or ischemic heart disease (*n* = 10) or from organ donors without cardiac disease (*n* = 15) whose hearts could not be used as grafts. The investigation conformed with the principles outlined in the Declaration of Helsinki, and the protocol was approved by the Ethics Committee of Helsinki University Central Hospital. All patients signed an informed consent form. The National Authority for Medicolegal Affairs (TEO) approved the use organ donor tissues.

### Statistical analysis

Values are reported as means ± s.e.m. Data were checked for normality and statistical analysis was performed with the two-tailed unpaired Student's *t*-test for two-group comparisons (for microarray and metabolomics statistical analysis see Supporting Information). For multiple group comparisons one-way ANOVA with the LSD or Dunnett's post-hoc test was used. For correlation between two continuous variables, linear regression with Spearman's rank test was used. The number of samples in each analysis is shown in the figures. Differences were considered statistically significant at *P < *0.05.

## Author contributions

RK, MB, MR and MT performed experiments, data acquisition, analysis and interpretation of data, statistical analyses, and wrote the manuscript; MRR performed metabolite uptake and tissue lipid experiments and commented on the manuscript; JMUS, AS, SH and JK performed PET analyses; JJH and AA performed protein biochemical analyses; MIM and JHL collected and provided human myocardial samples; LE performed electron microscopy; RH and VD performed vascular casting and electron microscopy; ZWZ and MS performed microCT imaging; EM supervised myocardial infarctions and echocardiography; KA designed experiments, conducted scientific direction and wrote the manuscript.

**The paper explained**

**Problem**

Ischemic heart disease is among the leading causes of death in the Western world. Despite intensive efforts, growth factors suitable for angiogenic gene therapy have not yet provided significant help in the treatment of cardiovascular disease. This is likely to change as we gain a better understanding of the underlying biology of these growth factors as well as of their regulation and functions. We therefore sought to elucidate the role of VEGF-B in the regulation of myocardial and vascular function in the heart, as well as its therapeutic potential.

**Results**

In this study, we show that transgenic expression of VEGF-B in the rat heart leads to a dramatic expansion of the coronary arterial tree and an increase in functional coronary reserve, accompanied by a shift in myocardial metabolism from fatty acid to glucose utilization. These changes led to favorable changes in cardiac function, both during ischemia and in old age. Importantly, VEGF-B levels were found to decrease in human cardiomyopathy, and the vascular and metabolic changes were reproducible using gene transfer to adult rats.

**Impact**

Our results indicate that VEGF-B could be used to enhance the coronary vasculature and to reprogram myocardial metabolism to improve cardiac function in ischemic heart disease. Our models also highlight the interaction between the endothelium and the cardiomyocytes. Indeed, the effects of vascular growth factors on metabolism as well as the overall interplay between angiogenesis, cell growth and metabolism are the focus of growing interest in the field.
