# Supplementary material for: VEGF-B-induced vascular growth leads to metabolic reprogramming and ischemia resistance in the heart
Source: EMBO Mol Med. 2014 Jan 21;6(3):307–21. doi: 10.1002/emmm.201303147 (PMC3958306; doi:10.1002/emmm.201303147)
Supplement: Supplementary file 1 [file emmm0006-0307-sd1.pdf]

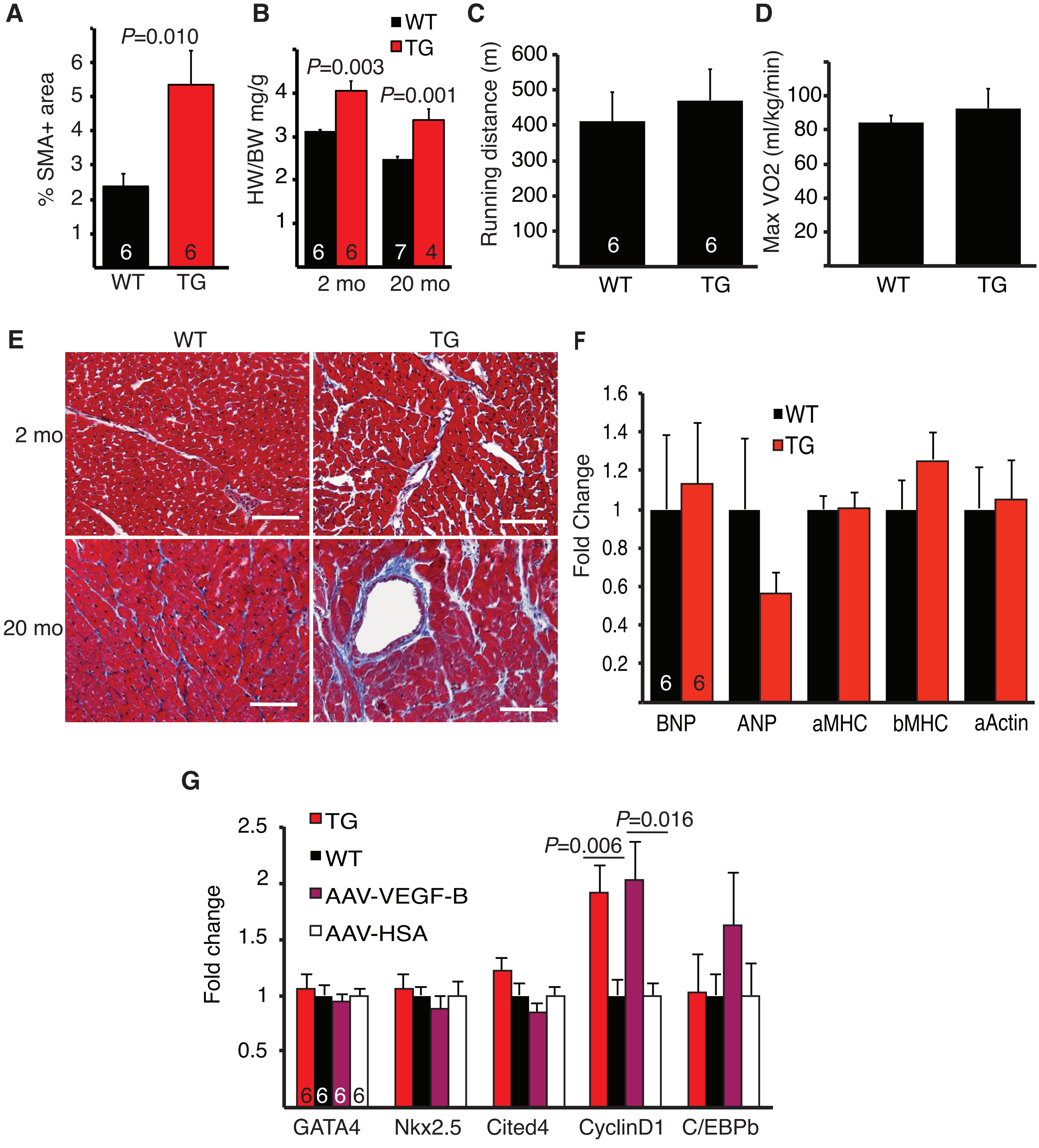

Supporting Information Figure 1. VEGF-B -induced hypertrophy remains physiological even in old rats. (A) SMA-positive (arterial) vessel area in hearts of pups at postnatal day 8. (B) Heart-to-body weight -ratios of young (2 months) and old (20-22 months) rats. **(**C) Running distance and (D) maximal oxygen consumption in the treadmill running test. (E) Representative Masson's trichrome stained heart sections showing increased fibrosis in old rats, but no difference between the WT and TG genotypes. (F) RNA expression of genes related to pathological hypertrophy. (G) RNA expression of transcription factors related to exercise-induced cardiac hypertrophy. Data is shown as mean ± S.E.M (Student's t-test), the number of animals in each experiment is indicated in the figures. Scale bars 50 µm.
