# Supplementary material for: VEGF-B-induced vascular growth leads to metabolic reprogramming and ischemia resistance in the heart
Source: EMBO Mol Med. 2014 Jan 21;6(3):307–21. doi: 10.1002/emmm.201303147 (PMC3958306; doi:10.1002/emmm.201303147)
Supplement: Supplementary file 2 [file emmm0006-0307-sd2.pdf]

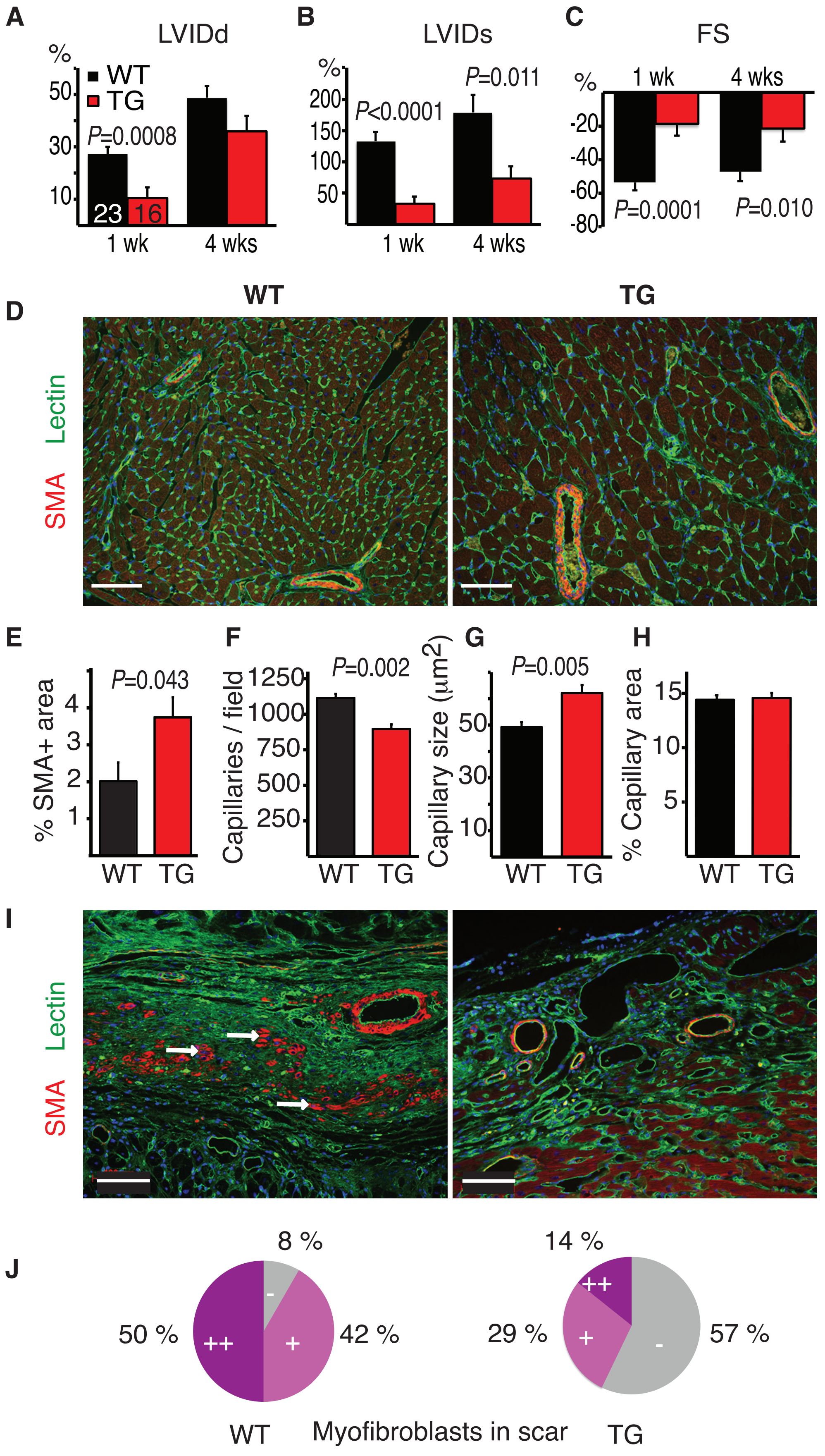

Supporting Information Figure 2. Increased vasculature and fewer myofibroblasts in the scar tissue in VEGF-B TG hearts after MI. (A-C) Changes in cardiac dimensions and function (% change from baseline) measured by echocardiography 1 and 4 weeks after MI (LVIDd/LVIDs = left ventricular internal diameter in diastole/systole, FS = fractional shortening) (D) Immunofluorescence staining of the remote myocardium for SMA (arteries) and lectin (endothelial cells). (E) Quantification of the SMA-positive vessel area percentage, (F) capillary density, (G) capillary size, and (H) and capillary area percentage. (I) SMA staining of myofibroblasts (arrows) in the scar tissue four weeks after MI. (J) Semi-quantitative analysis of the amount of myofibroblasts in the scar presented as the proportion of rats per genotype featuring: - (no), + (few) or ++ (several) myofibroblasts. Data is shown as mean ± S.E.M (Student's t-test). Scale bars 50 μm.
