# Supplementary material for: VEGF-B-induced vascular growth leads to metabolic reprogramming and ischemia resistance in the heart
Source: EMBO Mol Med. 2014 Jan 21;6(3):307–21. doi: 10.1002/emmm.201303147 (PMC3958306; doi:10.1002/emmm.201303147)
Supplement: Supplementary file 3 [file emmm0006-0307-sd3.pdf]

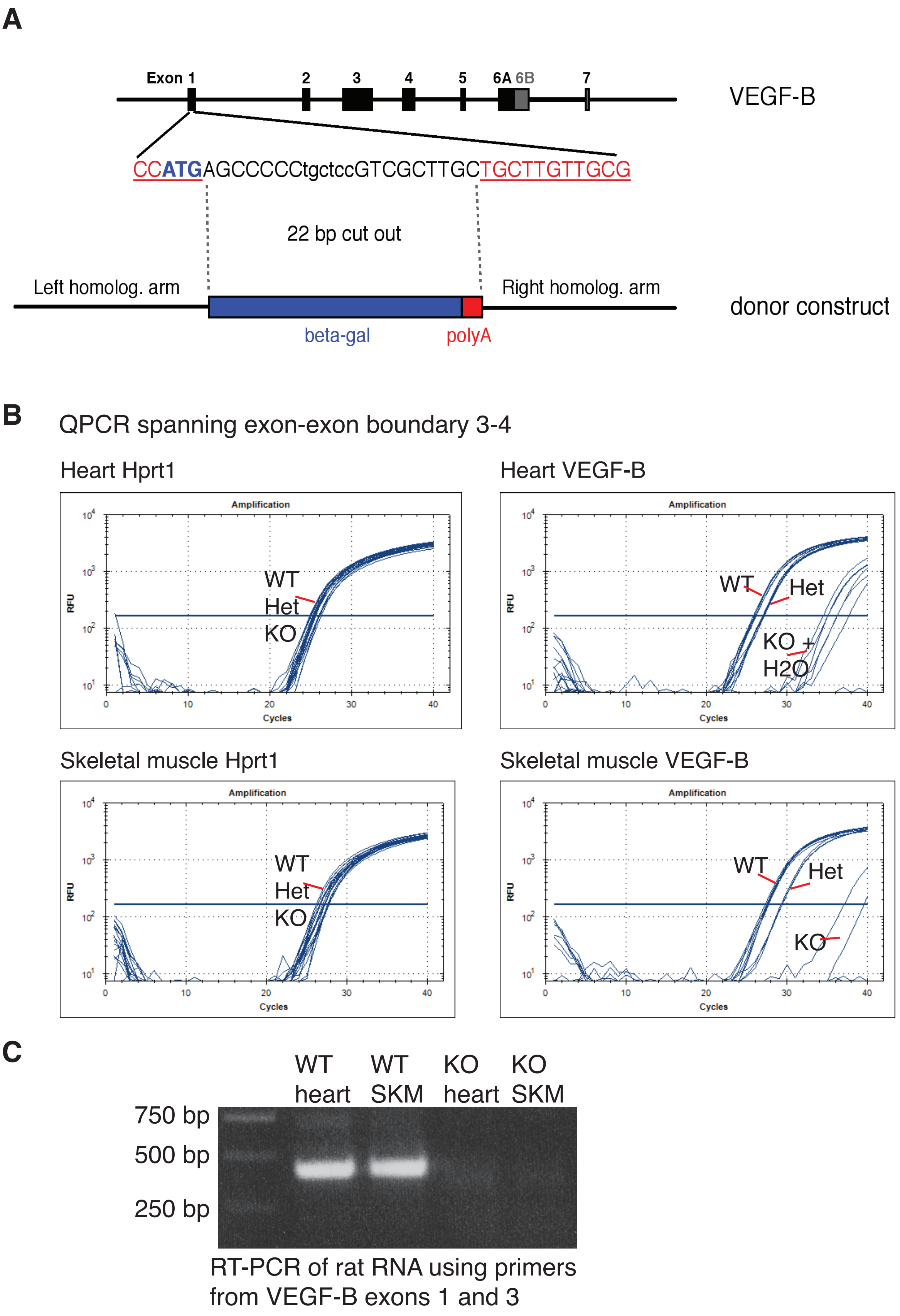

Supporting Information Figure 3. Analysis of VEGF-B RNA in homozygous and heterozygous KO rats. (A) Schematic of the VEGF-B deletion using zinc finger nucleases with beta-galactosidase knock-in. Validation of the deletion of VEGF-B transcript in KO rats with (B) quantitative real time RT-PCR using Hprt1 as a control gene, and (C) standard RT-PCR from the heart and skeletal muscle (SKM).
