# Supplementary material for: VEGF-B-induced vascular growth leads to metabolic reprogramming and ischemia resistance in the heart
Source: EMBO Mol Med. 2014 Jan 21;6(3):307–21. doi: 10.1002/emmm.201303147 (PMC3958306; doi:10.1002/emmm.201303147)
Supplement: Supplementary file 4 [file emmm0006-0307-sd4.pdf]

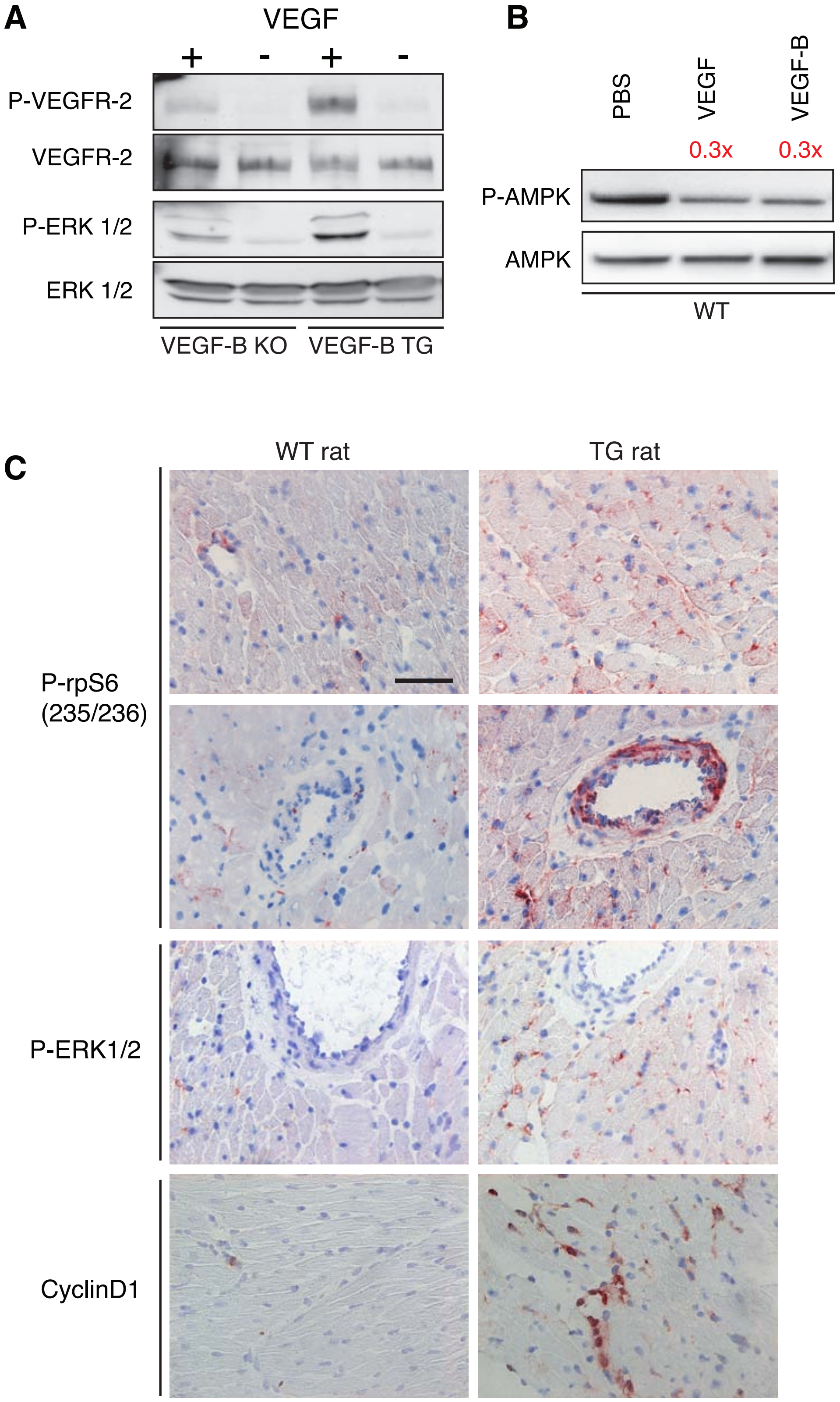

Supporting Information Figure 4. VEGF signal transduction in VEGF-B TG, KO and WT hearts. VEGF signal transduction in VEGF-B TG, KO and WT hearts. (A) Repeated experiment similar to that in Figure 4C. (B) AMPK phosphorylation in VEGF and VEGF-B stimulated hearts. Indicated in red is the P-AMPK signal intensity compared to the PBS control. (C) Staining of phosphorylated rpS6 and ERK1/2 in the WT and TG rat hearts. Note P-rpS6 staining in arterial smooth muscle cells, interstitial cells and cardiomyocytes in the TG hearts and P-ERK1/2 signal mainly in capillaries. The staining was stronger in the VEGF-B TG than in WT hearts in accordance with the Western blot results presented in Figure 4D. CyclinD1 was one of the most upregulated genes in the TG hearts. Immunostaining showed that it was highly expressed in endothelial cells in TG rats, but only a few positive cells were found in WT hearts. Scale bar 50 µm.
