# Supplementary material for: VEGF-B-induced vascular growth leads to metabolic reprogramming and ischemia resistance in the heart
Source: EMBO Mol Med. 2014 Jan 21;6(3):307–21. doi: 10.1002/emmm.201303147 (PMC3958306; doi:10.1002/emmm.201303147)
Supplement: Supplementary file 5 [file emmm0006-0307-sd5.pdf]

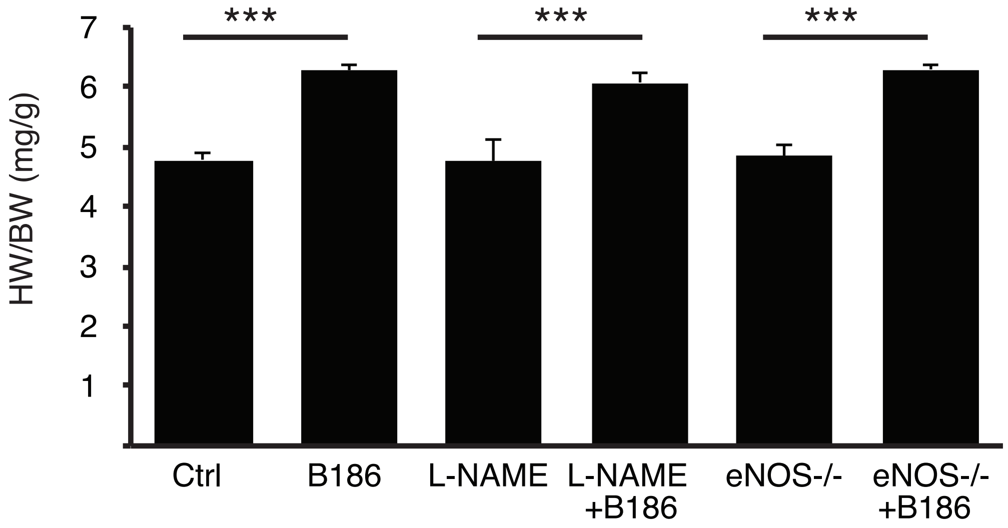

Supporting Information Figure 5. The VEGF-B-induced cardiac hypertrophy is not dependent on NOS activity. AAV-VEGF-B186 treatment for 4 weeks induced similar cardiac growth in mice treated with the NOS inhibitor L-NAME or deficient of eNOS as in the WT mice. Data is shown as mean ± S.E.M (one-way ANOVA with LSD post hoc test). ***P<.0001.
