# Supplementary material for: VEGF-B-induced vascular growth leads to metabolic reprogramming and ischemia resistance in the heart
Source: EMBO Mol Med. 2014 Jan 21;6(3):307–21. doi: 10.1002/emmm.201303147 (PMC3958306; doi:10.1002/emmm.201303147)
Supplement: Supplementary file 6 [file emmm0006-0307-sd6.pdf]

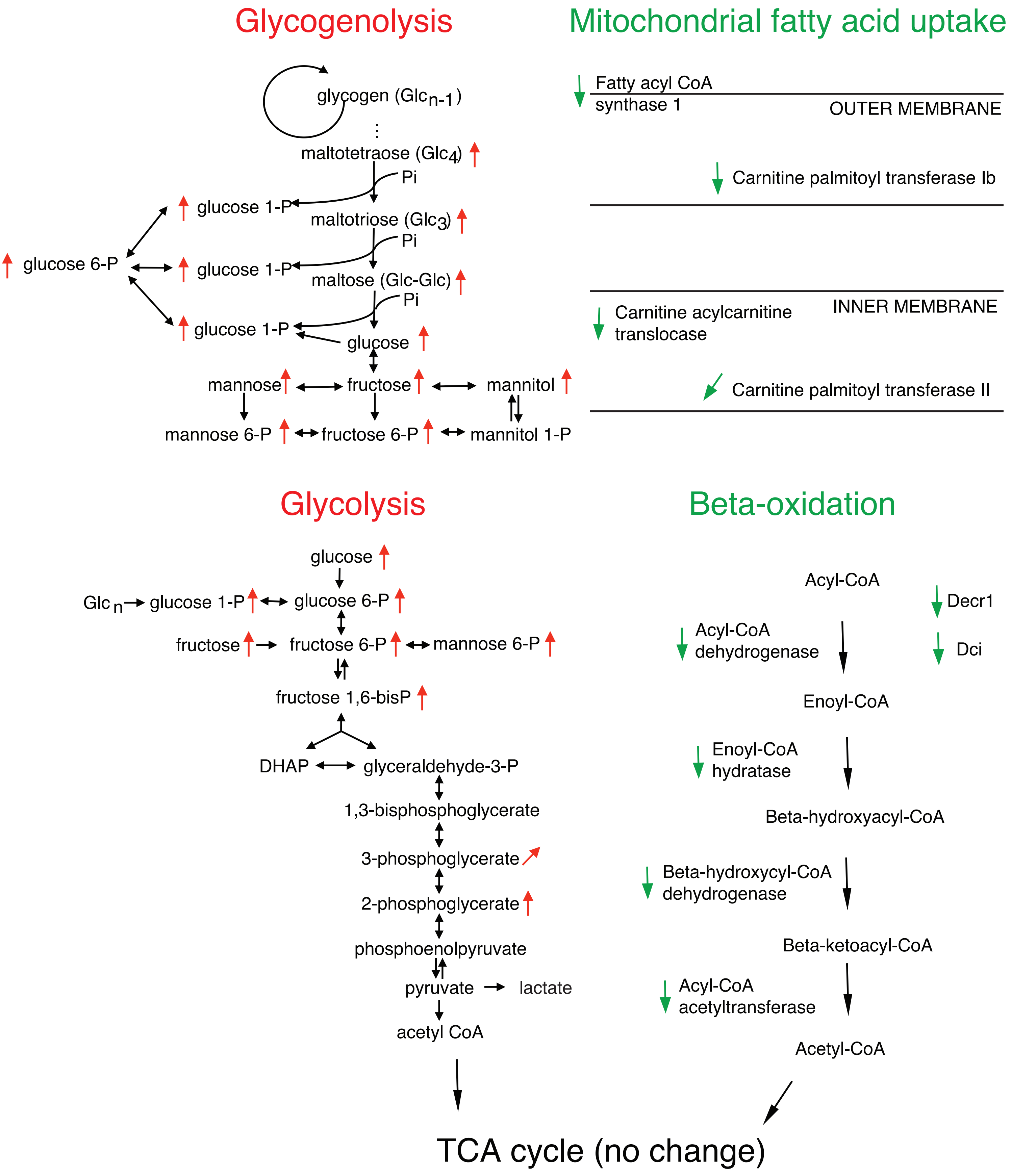

Supporting Information Figure 6. A schematic presentation of the metabolic shift from lipid oxidation to glucose utilization in the VEGF-B TG hearts. The main changes were increased levels of intermediates of glycogen and glucose breakdown in metabolomics analyses and reduced expression of genes responsible for mitochondrial fatty acid uptake and oxidation in microarray analysis of the VEGF-B TG hearts.
