# Supplementary material for: VEGF-B-induced vascular growth leads to metabolic reprogramming and ischemia resistance in the heart
Source: EMBO Mol Med. 2014 Jan 21;6(3):307–21. doi: 10.1002/emmm.201303147 (PMC3958306; doi:10.1002/emmm.201303147)
Supplement: Supplementary file 7 [file emmm0006-0307-sd7.pdf]

Supporting Information Table 1. **Echocardiography of cardiac dimensions of 20-22-month-old VEGF-B TG and WT rats.** EF, ejection fraction; FS, fractional shortening; SV, stroke volume; EDV, end-diastolic volume; ESV, end-systolic volume; LVIDs, left ventricular internal diameter in systole; LVIDd, left ventricular internal diameter in diastole; LVPWTs, left ventricular posterior wall thickness in systole; LVPWTd, left ventricular posterior wall thickness in diastole; Data is shown as mean ± S.E.M (Student's t-test); * $P<.05$; $N=4+7$.

	**WT**	**TG**
EF (%)	88.18 ± 2.07	87.22 ± 3.17
FS (%)	54.58 ± 3.51	53.41 ± 4.77
SV (ml)	0.53 ± 0.02	0.71 ± 0.07 *P=.009
EDV (ml)	0.61 ± 0.02	0.83 ± 0.09 * P=.016
ESV (ml)	0.07 ± 0.01	0.11 ± 0.04
LVIDs (mm)	2.91 ± 0.24	3.34 ± 0.36
LVIDd (mm)	6.39 ± 0.08	7.13 ± 0.29 * P=.013
LVPWTs (mm)	3.34 ± 0.17	3.23 ± 0.21
LVPWTd (mm)	2.01 ± 0.11	1.96 ± 0.11

Supporting Information Table 2. Significantly (FDR<.05) up- and downregulated genes in both VEGF-B TG and AAV-VEGF-B hearts. Separate Dataset as .xls file.
