# Supplementary material for: VEGF-B-induced vascular growth leads to metabolic reprogramming and ischemia resistance in the heart
Source: EMBO Mol Med. 2014 Jan 21;6(3):307–21. doi: 10.1002/emmm.201303147 (PMC3958306; doi:10.1002/emmm.201303147)
Supplement: Supplementary file 9 [file emmm0006-0307-sd9.pdf]

EMBO Molecular Medicine

VEGF-B-induced vascular growth leads to metabolic reprogramming and ischemia resistance in the heart

Riikka Kivelä, Maija Bry, Marius R. Robciuc, Markus Räsänen, Miia Taavitsainen, Johanna M.U. Silvola, Antti Saraste, Juha J. Hulmi, Andrey Anisimov, Mikko I. Mäyränpää, Jan H. Lindeman, Lauri Eklund, Sanna Hellberg, Ruslan Hlushchuk, Zhen W. Zhuang, Michael Simons, Valentin Djonov, Juhani Knuuti, Eero Mervaala and Kari Alitalo

Corresponding author: Kari Alitalo, Biomedicum Helsinki Faculty of Medicine

---

Review timeline:

Submission date:	08 June 2013
Editorial Decision:	08 July 2013
Revision received:	11 November 2013
Editorial Decision:	28 November 2013
Revision received:	05 December 2013
Accepted:	06 December 2013

Transaction Report:

(Note: With the exception of the correction of typographical or spelling errors that could be a source of ambiguity, letters and reports are not edited. The original formatting of letters and referee reports may not be reflected in this compilation.)

Editor: Roberto Buccione

1st Editorial Decision 08 July 2013

Thank you for the submission of your manuscript to EMBO Molecular Medicine. We have now heard back from the three Reviewers whom we asked to evaluate your manuscript.

You will see that while Reviewer 3 is quite supportive of your work, Reviewers 1 and 2 express a number of concerns that prevent us from considering publication at this time. I will not dwell into much detail, as the evaluations are detailed and self-explanatory and just mention a few main points.

Reviewer 1 suggests that appropriate experimentation is required to clarify whether infarct size and areas at risk are comparable between strains. S/he would also like to see data supporting the claim that excess VEGF-B allowed more VEGF to bind VEGFR-2. Reviewer 1 lists other important issues that require your action. This Reviewer also notes that a therapeutic approach in stressed hearts (or alternatively to show that the pathways are affected in human tissue) is necessary to demonstrate the translational potential of the findings. All in all, Reviewer 1 is specifically concerned about the therapeutic value of the findings. This is especially important for EMBO Molecular Medicine and I would therefore ask you to place a special effort in this respect.

Reviewer 2 recognises that the involvement of VEGFR2 is a novel element but would like to see evidence for the suggestion that VEGFR2 inhibition does not affect VEGFR1-dependent arteriolar growth. S/he would also like to see if the vascular and hypertrophic growth patterns and the ischemia resistance induced by VEGF-B depend on the observed metabolic changes. Reviewer 2 also lists other issues that require your action.

Reviewer 3 would like you to include, as supplementary information, the expression analysis of genes associated with pathological remodelling.

Considered all the above, while publication of the paper cannot be considered at this stage, we would be prepared to consider a substantially revised submission, with the understanding that the Reviewers' concerns must be fully addressed, with additional experimental data where appropriate and that acceptance of the manuscript will entail a second round of review.

Please note that it is EMBO Molecular Medicine policy to allow a single round of revision only and that, therefore, acceptance or rejection of the manuscript will depend on the completeness of your responses included in the next, final version of the manuscript.

As you know, EMBO Molecular Medicine has a "scooping protection" policy, whereby similar findings that are published by others during review or revision are not a criterion for rejection. However, I do ask you to get in touch with us after three months if you have not completed your revision, to update us on the status. Please also contact us as soon as possible if similar work is published elsewhere.

I look forward to seeing a revised form of your manuscript as soon as possible.

***** Reviewer's comments *****

Referee #1 (Remarks):

Kivelä and colleagues studied the role of VEGF-B in cardiac metabolism and suggest a therapeutic potential for their findings. Although the observed phenotype is striking and therapeutic potential might be present, this is not yet clear from the current experiments.

Major comments:

- Already at baseline, these animals have an increased coronary vasculature are there any difference in cardiac contractile performance before surgery. Is there a difference in number of arterioles per single cardiomyocyte?
- Upon MI, IS is reduced as % of the LV at 4 weeks, but are IS and area at risks (AAR) comparable upon acute MI since the perfusion structure is different between the strains. For this, TTC staining and evans blue dye infusion experiments are essential at e.g. 24 or 48 hours. Differences in these areas could explain downstream effects on oxygen consumption and fibrosis as well.
- "To study the arterial and metabolic phenotype" (page 7) as stated by the authors, but not clear why they make this statement, since they only demonstrated the perfusion defect. In the same paragraph they refer to echographic data that could not be found in the manuscript ?
- Not clear what the mechanism is of the VEGF-B overexpression in myocytes, the increased growth in arterial bed and subsequent hypertrophic myocyte response? What are normal levels of VEGF-B in cardiomyocytes and what is the level of upregulation in the TG and AAV approach? The use of the VEGF-B KO animals is not a myocyte specific approach, so redundant for the understandings. Not providing detailed analysis as well would suggest to remove this part of the paper.
- How did the inflammatory response differ in the acute setting of MI, on long term (assumed since this was not clearly stated in the paper and figures not presented) there was no difference.
- Relative few genes were up- and down regulated, compared to the observed massive phenotypes? How can the authors explain this?
- Page 10: "suggesting that an excess of VEGF-B allowed more VEGF to bind to and activate VEGFR-2", this is purely speculative and need more mechanistic follow-up.
- Metabolic shift that is now studied in vivo, should be accompanied by analysis in stressed hearts and a more detailed analysis in vitro. In which a better controlled environment could be mimicked/studied to see the myocyte differences because shifts in metabolic usage could be explained by several mechanisms.
- Since other rapports demonstrated an effect of cardiomyocyte apoptosis, this should be included in the short term analysis as indicated above.
- From current data, it is not clear whether the increased glycolysis is direct effect of the TG or just a

consequence of the better perfused hearts and therefore more healthy tissue and oxygen supply. As indicated in the last part of the discussion, the therapeutic potential is a novel idea but should be tested in a cardiac stress model.

Minor comments:

- Please provide Maximal exercise capacity in a figure (page 6)
- The readability of the paper would definitely improve by using chronological order and correct referring of the figures in the text. This should be improved.
- Upon AAV injection, cardiomyocytes did not show a significant hypertrophic response, please remove the suggestive sentence (tended). Please provide a similar quantification of the vessel diameters as in fig 1, this would allow a direct comparison of the approaches.
- Supple 5 A, please provide a quantification for the different stainings.
- "Gene Set Enrichment Analysis (GSEA) of unfiltered data from both the TG and AAV sample sets confirmed this result (Fig. 6A, B) (page 12)." Please provide a single heat map and not two different figures, that will allow a better comparison of individual genes.
- Discussion should be shortened.

Referee #2 (Comments on Novelty/Model System):

The authors are able to show a vector-based VEGF-B therapy which significantly decreases the extent of myocardial infarction without serious side effects, which are known for e.g. VEGF-A. The therapeutic approach is backed by transgene and knockout rat experiments. Moreover, meticulous metabolic analysis revealed a novel sigature trait of VEGF-B treated hearts: a switch from lipolsys to more glycolytic metabolism

Referee #2 (Remarks):

The manuscript of Kivelae, Alitalo et al. describes the role of VEGF-B in vascular growth and metabolic alteration s of the ischemic heart. To this purpose, cardiomyocyte specific VEGF-B transgene and knockout rats and recombinant adeno-associated (rAAV)-mediated VEGF-B overexpression were used.
The authors reveal an increase in arteriogenesis , in particular of the small arterioles (<100µm diameter). 4 weeks after induction of myocardial infarction by permanent LAD ligation, infarct size was smaller, whereas perfusion and oxygen consumption of the infarcted and border zone areas were larger in the VEGF-B transgenic rat hearts. Capillary diameters were found larger, but capillary per cardiomyocyte ratio was not increased two months after systemic rAAV-VEGF-B transduction.
With respect to angiogenic signaling, Notch signaling as well as Erk1/2/Akt/mTorc1 pathways were found activated after VEGF-B exposure. Blocking VEGFR2 with a soluble VGEFR2-rAAV blocked VEGF-B-mediated capillary enlargement, but not hypertrophy. Metabolically, VEGF-B enhancement reduces fatty acid degradation, whereas fatty acid synthase was specifically increased. Fatty acid uptake, however, appeared unaltered among wildtype and transgenic as well as knockout hearts and peripheral muscles, although Fatp4 was increased in VEGF-B transgenic hearts.
This interesting and well-organized manuscript extends previous papers focusing on VEGF-B induced vascular growth in hearts subjected to chronic coronary occlusion. Here, a particular protection is provided by VEGF-B. On the other hand, the interplay of vascular growth, hypertrophy and metabolism renders some issues to be further clarified.
#1. The main vascular effect of VEGF-B (transgenic or rAAV-transduced) on the vascular tree is arteriolar and arterial growth, which yields a distinct protection against coronary occlusion, most likely due to improved collateralization (Fig.1&2). Is the same extent of cardioprotection provided by rAAV-VEGF-B or is a step provided by VEGF-B overexpressio during embryonic development essential?
#2. The involvement of VEGFR2 is a novel signaling element, since in former papers, mostly VEGFR1 seemed involved (Bry et al., Circ 2010; Zentilin et al., FASEB J 2010). Of note, VEGFR2 is involved only in vascular growth without affecting hypertrophy, which was previously correlated to VEGFR1 signaling. Since the authors show that VEGFR2 is not present in the coronary arteries, but expressed in capillaries, this lateralization suggests that VEGFR2 inhibition does not affect the

VEGFR1-dependent arteriolar growth. Is there evidence for this suggestion?
#3. The authors show that fatP4 is increased (Fig.6F), but question its role as a fatty acid importer. Is the result of Fig.7 (uptake of radioactive-labeled fatty acids) also present after shorter exposure of VEGF-B (e.g. rAAV-VEGF-B for 14 d?)
#4. Along the same lines, is the vascular and hypertropohic growth pattern as well as the ischemia resistance induced by VEGF-B depending on the metabolic changes the authors observed (enhanced glucose catabolization and decreased lipid oxidation)? Moreover, is the the cardioprotection provided in Fig.2 requiring the metabolic alterations?

Minor:

#1: Suppl. Fig. 2 contains information which should be displayed in the main paper.

Referee #3 (Remarks):

This is an excellent article describing the effects of VEGF-B overexpression or deficiency in the adult rat heart. The authors used cardiomyocyte-specific transgene expression, gene targeting, or virus-mediated gene transfer to overexpress or to inactivate the VEGF-B gene in rats, and studied the effects of this manipulation in physiological and pathological conditions. They show that VEGF-B TG rats develop enhanced functional coronary vasculature, resulting in protection of the heart from ischemic damage. On the other hand, hearts of VEGF-B deficient rat had a normal vasculature. AAV-mediated VEGF-B overexpression had similar effects as the VEGF-B transgene. Then the authors go on to study the mechanisms of action of VEGF-B in the heart. They show that VEGF-B increases VEGF-A signaling via VEGFR-2, identify downstream signaling pathways and report metabolic changes indicative of a reprogramming of cardiomyocyte metabolism. The authors conclude that VEGF-B could be used to improve cardiac function in ischemic heart disease.

The results are convincing and support the conclusions drawn. Overall, the study is of high technical quality and has important medical implications. Similar studies were previously performed in mice, however, as pointed out by the authors, certain effects such as arteriogenesis occured only in rats. This finding may turn out to be relevant for therapy. The analysis of the mechanisms of VEGF-B action provides novel and helpful information.

I have only a minor point of criticism:

- the results of the expression analysis of genes associated with pathological remodeling, referred to as data not shown (page 7), should be included as supplementary information.

1st Revision - authors' response 11 November 2013

We thank the reviewers for their constructive criticism and valuable comments. We have addressed these below point by point.

In addition, as the editor suggested, we have also acquired human samples to evaluate clinical significance of our findings. VEGF-B expression was significantly decreased in ischemic and dilated heart disease in humans in two separate data sets (one from Leiden and one from Helsinki). This data has been added to the manuscript.

Comments from Reviewer #1:

Kivelä and colleagues studied the role of VEGF-B in cardiac metabolism and suggest a therapeutic potential for their findings. Although the observed phenotype is striking and therapeutic potential might be present, this is not yet clear from the current experiments.

Major comments:

Already at baseline, these animals have an increased coronary vasculature are there any difference in cardiac contractile performance before surgery. Is there a difference in number of arterioles per single cardiomyocyte?

The contractile performance in young rats has been studied in our previous publication, Bry et al. Circulation 2010. There were no differences in the ejection fraction or fractional shortening between the TG and WT rats. This was also the case in the old (20-22 mo) rats presented in this study. The number of arterioles in TG rats is increased 2-3 fold depending on their size (2D microCT quantification). Since the increase in cardiomyocyte size and heart weight was about 20-30% (Bry et al, 2010), this indicated an increased arteriole/cardiomyocyte-ratio. An earlier report has also shown that VEGF-B does not induce CMC proliferation (Zentilin et al. 2010). Thus, the arteriole/CMC – ratio is increased in TG rats, which has now been added to the results section on page 6.

Upon MI, IS is reduced as % of the LV at 4 weeks, but are IS and area at risks (AAR) comparable upon acute MI since the perfusion structure is different between the strains. For this, TTC staining and evans blue dye infusion experiments are essential at e.g. 24 or 48 hours. Differences in these areas could explain downstream effects on oxygen consumption and fibrosis as well.

This is an important point. We have now included an additional MI experiment, where although the area-at-risk was not significantly changed, the TG hearts tended to have smaller infarct areas already 24h after MI, indicating that the VEGF-B –induced effects on vessel remodelling and metabolism protected the cardiomyocytes from ischemia. This has been added to page 8 of the Results and Discussion and to Figure 2(H and I).

"To study the arterial and metabolic phenotype" (page 7) as stated by the authors, but not clear why they make this statement, since they only demonstrated the perfusion defect. In the same paragraph they refer to echographic data that could not be found in the manuscript ?

We apologize for the confusing statement, which we have now revised accordingly as: "To study if the increased vasculature seen in the VEGF-B TG rats provides protection from ischemic myocardial damage, TG and WT rats were subjected to experimental myocardial infarction (MI)". We have also included the echocardiographic data in Supp. Figure 2. This data was not included in the previous version, as the cardiac performance in younger rats has already been published.

Not clear what the mechanism is of the VEGF-B overexpression in myocytes, the increased growth in arterial bed and subsequent hypertrophic myocyte response? What are normal levels of VEGF-B in cardiomyocytes and what is the level of up regulation in the TG and AAV approach? The use of the VEGF-B KO animals is not a myocyte specific approach, so redundant for the understandings. Not providing detailed analysis as well would suggest to remove this part of the paper.

The endogenous expression of VEGF-B among different tissues is the highest in the heart (cardiomyocytes). This has now been mentioned in the Introduction (page 3). The level of up regulation varies between the transgenic lines and due to the transduction efficiencies in AAV experiments (20-100 fold increase in mRNA expression, we do not have working antibody for rat endogenous VEGF-B to calculate the level of up regulation) From a therapeutic point of view it is important that, in contrast to other VEGF family members, even high amounts of VEGF-B are safe, whereas high levels of e.g. VEGF-A and VEGF-C are toxic, thus providing a much smaller therapeutic window. This has now been added to the discussion on page 21.

© EMBO

This is the first model of VEGF-B gene deletion in rats, and we believe this result should be included when considering VEGF-B as a therapeutic molecule. The analysis of myocardial infarctions in KO rats is not as extensive as in TG rats, as no significant differences could be found. It is not conceivable at this time to create a cell-specific conditional VEGF-B KO model in rats. The main reason for including this model in our manuscript was to compare the metabolic effects to those in the TG and WT rats, and also to previously published findings in KO mice, which we were not able to reproduce.

How did the inflammatory response differ in the acute setting of MI, on long term (assumed since this was not clearly stated in the paper and figures not presented) there was no difference.

We have now included analysis of both acute and long-term inflammatory response after MI. In the infarct/border zone there were no differences between the genotypes, but CD68 was up regulated in the TG remote myocardium compared to WT four weeks after MI. However, at 24h there was no differences. This has been added to the Results (page 8).

Relative few genes were up- and down regulated, compared to the observed massive phenotypes? How can the authors explain this?

The number of affected genes depends on the filtering criteria in the bioinformatics analyses. Using the FDR value <0.05 there were 2012 genes changed, with FDR <0.01 1151 genes and with FDR<0.001 522 genes in TG versus WT hearts. In AAV-studies, the numbers were a bit lower, but the affected genes were very similar as shown in the manuscript. For the DAVID analyses, we used stringent criteria to avoid false positive results. In GSEA analyses we have used the whole normalized data set without any set cut-off criteria beforehand. With both approaches, similar pathways and gene clusters were obtained, confirming the findings.

Page 10: "suggesting that an excess of VEGF-B allowed more VEGF to bind to and activate VEGFR-2", this is purely speculative and need more mechanistic follow-up.

This mechanism has now been published by Anisimov et al. in Science Signaling (The basis for the distinct biological activities of vascular endothelial growth factor receptor-1 ligands, July 2013). These results showed that VEGF-B does not activate VEGFR-2, and that mode of binding of VEGF-B to VEGFR-1 differs significantly from that of PlGF (and VEGF).

Metabolic shift that is now studied in vivo, should be accompanied by analysis in stressed hearts and a more detailed analysis in vitro. In which a better controlled environment could be mimicked/studied to see the myocyte differences because shifts in metabolic usage could be explained by several mechanisms.

This is also a very important point. We have now performed an ischemia/reperfusion experiment together with mitochondrial function analyses using different substrates. These results provide strong evidence that VEGF-B protects cardiomyocyte mitochondria from I/R stress, since mitochondrial complex I activity was significantly improved in ischemic TG hearts compared to WT hearts (no difference in the basal state). Complex I function is well known to be affected in ischemic heart diseases and heart failure.

Since other rapports demonstrated an effect of cardiomyocyte apoptosis, this should be included in the short term analysis as indicated above.

This is related to the previous question and answer, and we believe that the mitochondrial protection seen in our study explains the reduced apoptosis seen in earlier studies.

From current data, it is not clear whether the increased glycolysis is direct effect of the TG or just a consequence of the better perfused hearts and therefore more healthy tissue and oxygen supply. As indicated in the last part of the discussion, the therapeutic potential is a novel idea

but should be tested in a cardiac stress model.

It is clear from our results that VEGF-B affects both the vasculature as well as cardiomyocytes, where the former occurs mainly via enhanced VEGF signalling through VEGFR-2, and the latter via VEGF-B–VEGFR-1 signalling. Whether the metabolic changes are direct effects of VEGF-B on metabolic pathways or secondary effects of cardiomyocyte growth, is very difficult to distinguish. Our short term AAV-experiments suggest that the vasculature effects come first and metabolic effects follow later, as we did not see metabolic changes after 2 weeks of VEGF-B expression, when the angiogenic response was already detectable. However, our data from both the infarcted hearts and the old rats support the idea that increased glucose oxidation is rather favourable than detrimental to the heart. This was further supported by the added I/R experiment showing protection from mitochondrial damage.

Minor comments:

Please provide Maximal exercise capacity in a figure (page 6)

This has now been added to the Supplemental Figure 1C, and D.

The readability of the paper would definitely improve by using chronological order and correct referring of the figures in the text. This should be improved.

We have now revised the order of the panels in the Figures and corresponding text in results to improve the readability.

Upon AAV injection, cardiomyocytes did not show a significant hypertrophic response, please remove the suggestive sentence (tended).

We thank the reviewer for this comment; the sentence has now been revised accordingly.

Please provide a similar quantification of the vessel diameters as in fig 1, this would allow a direct comparison of the approaches.

We have now included this quantification in Fig. 4.

Supple 5 A, please provide a quantification for the different stainings.

Quantification for pS6 and pERK has been performed with Western blotting, which we believe to be more reliable than quantification from IHC. IHC was used to show the localization (now SFig. 4C). Quantification for cyclinD1 was done from mRNA (shown in SFig.1)

"Gene Set Enrichment Analysis (GSEA) of unfiltered data from both the TG and AAV sample sets confirmed this result (Fig. 6A, B) (page 12)." Please provide a single heat map and not two different figures, that will allow a better comparison of individual genes.

This figure has now been revised accordingly.

Discussion should be shortened.

The discussion has now been shortened. However, this is an extensive analysis and it is necessary to discuss the many important (and also some conflicting) results in this paper.

Referee #2 (Comments on Novelty/Model System):

The authors are able to show a vector-based VEGF-B therapy which significantly decreases the extent of myocardial infarction without serious side effects, which are known for e.g. VEGF-A.

The therapeutic approach is backed by transgene and knockout rat experiments. Moreover, meticulous metabolic analysis revealed a novel sigature trait of VEGF-B treated hearts: a switch from lipolsys to more glycolytic metabolism

Referee #2 (Remarks):

The manuscript of Kivelae, Alitalo et al. describes the role of VEGF-B in vascular growth and metabolic alteration s of the ischemic heart. To this purpose, cardiomyocyte specific VEGF-B transgene and knockout rats and recombinant adeno-associated (rAAV)-mediated VEGF-B overexpression were used.

The authors reveal an increase in arteriogenesis , in particular of the small arterioles (<100um diameter). 4 weeks after induction of myocardial infarction by permanent LAD ligation, infarct size was smaller, whereas perfusion and oxygen consumption of the infarcted and border zone

areas were larger in the VEGF-B transgenic rat hearts. Capillary diameters were found larger, but capillary per cardiomyocyte ratio was not increased two months after systemic rAAV-VEGF-B transduction.

With respect to angiogenic signalling, Notch signalling as well as Erk1/2/Akt/mTorc1 pathways were found activated after VEGF-B exposure. Blocking VEGFR2 with a soluble VGEFR2-rAAV blocked VEGF-B-mediated capillary enlargement, but not hypertrophy. Metabolically, VEGF-B enhancement reduces fatty acid degradation, whereas fatty acid synthase was specifically increased. Fatty acid uptake, however, appeared unaltered among wild type and transgenic as well as knockout hearts and peripheral muscles, although Fatp4 was increased in VEGF-B transgenic hearts.

This interesting and well-organized manuscript extends previous papers focusing on VEGF-B induced vascular growth in hearts subjected to chronic coronary occlusion. Here, a particular protection is provided by VEGF-B. On the other hand, the interplay of vascular growth, hypertrophy and metabolism renders some issues to be further clarified.

#1. The main vascular effect of VEGF-B (transgenic or rAAV-transduced) on the vascular tree is arteriolar and arterial growth, which yields a distinct protection against coronary occlusion, most likely due to improved collateralization (Fig.1&2). Is the same extent of cardioprotection provided by rAAV-VEGF-B or is a step provided by VEGF-B overexpressio during embryonic development essential?

We thank the reviewer for the constructive comments. AAV-VEGF-B was able to induce arteriogenesis similarly to what was seen in transgenic rats, although not to the same extent, indicating that overexpression during embryonic development is not essential, but rather that the expression level and duration makes the difference. VEGF-B is highly expressed in the heart endogenously, but not required for embryonic or postnatal development. The phenotype in TG rats is stronger than in AAV-VEGF-B rats, but the phenotype and gene expression changes are very similar. The AAV-VEGFB -induced arterial expansion analysed by microCT has now been included (Fig. 4) similarly as the TG data in Fig.1.

#2. The involvement of VEGFR2 is a novel signalling element, since in former papers, mostly VEGFR1 seemed involved (Bry et al., Circ 2010; Zentilin et al., FASEB J 2010). Of note, VEGFR2 is involved only in vascular growth without affecting hypertrophy, which was previously correlated to VEGFR1 signalling. Since the authors show that VEGFR2 is not present in the coronary arteries, but expressed in capillaries, this lateralization suggests that VEGFR2 inhibition does not affect the VEGFR1-dependent arteriolar growth. Is there evidence for this suggestion?

We have now analysed the expression of Dll4 as an arterial marker in the sVEGFR-2 experiment. AAV-VEGF-B induced a significant increase in Dll4 expression compared to the empty vector, but when combined with AAV-sVEGFR-2 there was no significant change, indicating the involvement of VEGFR-2 also in the VEGF-B –induced arteriogenesis. It has been suggested that there is signalling between capillaries and their proximal arteries and also a role for VEGFR-2 in arteriogenesis has been reported (e.g. Lanahan AA, DevCell 2010). This result has been added to the text on page 12.

#3. The authors show that fatP4 is increased (Fig.6F), but question its role as a fatty acid importer. Is the result of Fig.7 (uptake of radioactive-labelled fatty acids) also present after shorter exposure of VEGF-B (e.g. rAAV-VEGF-B for 14 d?)

We have now analysed both fatty acid and glucose uptake after 14 days of AAV-VEGF-B expression and did not observe any significant differences between VEGF-B transduced and control rats. At this time point there were also no changes in Fatp4 or other metabolic gene expressions yet, together suggesting that metabolic changes develop later than the vascular effects. This result has been added to page 14.

#4. Along the same lines, is the vascular and hypertropohic growth pattern as well as the ischemia resistance induced by VEGF-B depending on the metabolic changes the authors observed (enhanced glucose catabolization and decreased lipid oxidation)? Moreover, is the cardioprotection provided in Fig.2 requiring the metabolic alterations?

We have shown that the vascular and hypertrophic changes occur prior to metabolic changes (Results, pages 14-15). The metabolic alterations are very complex, thus it is not conceivable that we can dissect their role in the cardioprotection in vivo. However, it is known that activation of fatty acid metabolism actually results in contractile failure in the hypertrophied rat heart, whereas drugs favouring glucose oxidation can protect the ischemic heart. This discussion has been elaborated on page 19-20.

Minor:

#1: Suppl. Fig. 2 contains information which should be displayed in the main paper.

Based on the comments of reviewer #1, we have performed additional MI and ischemia/reperfusion experiments, which are now included in the manuscript. Thus, we believe that the data presented in SFig.2 is secondary to the changes observed. However, we are willing to move some data if the reviewer still finds it necessary.

<u>Referee #3 (Remarks):</u>

This is an excellent article describing the effects of VEGF-B overexpression or deficiency in the adult rat heart. The authors used cardiomyocyte-specific transgene expression, gene targeting, or virus-mediated gene transfer to overexpress or to inactivate the VEGF-B gene in rats, and studied the effects of this manipulation in physiological and pathological conditions. They show that VEGF-B TG rats develop enhanced functional coronary vasculature, resulting in protection of the heart from ischemic damage. On the other hand, hearts of VEGF-B deficient rat had a normal vasculature. AAV-mediated VEGF-B overexpression had similar effects as the VEGF-B transgene. Then the authors go on to study the mechanisms of action of VEGF-B in the heart. They show that VEGF-B increases VEGF-A signalling via VEGFR-2, identify downstream signalling pathways and report metabolic changes indicative of a reprogramming of cardiomyocyte metabolism. The authors conclude that VEGF-B could be used to improve cardiac function in ischemic heart disease.

The results are convincing and support the conclusions drawn. Overall, the study is of high technical quality and has important medical implications.

Similar studies were previously performed in mice, however, as pointed out by the authors, certain effects such as arteriogenesis occurred only in rats. This finding may turn out to be relevant for therapy. The analysis of the mechanisms of VEGF-B action provides novel and helpful information.

I have only a minor point of criticism:

- the results of the expression analysis of genes associated with pathological remodelling, referred to as data not shown (page 7), should be included as supplementary information.

We thank the reviewer for the encouraging comments. The suggested data have now been included in Supplementary Figure 1.

2nd Editorial Decision 28 November 2013

Thank you for the submission of your revised manuscript to EMBO Molecular Medicine. We have now received the enclosed reports from the Reviewers who were asked to re-assess it. As you will see the Reviewers are now fully supportive and I am pleased to inform you that we will be able to accept your manuscript pending the following final minor amendments and requests:

1) As per our Author Guidelines, the description of all reported data that includes statistical testing must state the name of the statistical test used to generate error bars and P values, the number (n) of independent experiments underlying each data point (not replicate measures of one sample), and the

actual P value for each test (not merely 'significant' or 'P < 0.05').

2) Please provide higher resolution images for Figures 5 and 7. I refer specifically to the blots in Figure 5D (bottom 5 strips) and Fig. 7D. You will notice that the protein bands become noticeably blocky when you zoom in.

3) We would need a short list (up to 5) of bullet points that summarize the key NEW findings in your work. The bullet points (in the third person) should be designed to be complementary to the abstract and will be used online in our new platform (coming January 2014).

4) We are now encouraging the publication of source data, particularly for electrophoretic gels and blots, with the aim of making primary data more accessible and transparent to the reader. Would you be willing to provide a PDF file per figure that contains the original, uncropped and unprocessed scans of all or at least the key gels used in the manuscript? The PDF files should be labeled with the appropriate figure/panel number, and should have molecular weight markers; further annotation may be useful but is not essential. The PDF files will be published online with the article as supplementary "Source Data" files. If you have any questions regarding this just contact me.

Finally, I thought you might like to know that I have commissioned a Closeup article on your work. Closeups are short commentaries (our version of News and Views) designed to emphasize the novelty and broader implications of selected articles. We will be including the Closeup piece and your article in the same issue (to be determined).

I look forward to receiving the final version of your manuscript as soon as possible.

***** Reviewer's comments *****

Referee #1 (Remarks):

I would like to thank the authors for adressing point-to-point my comments in detail and think the manuscript improved drastically. I have no further comments.

Referee #2 (Remarks):

The revised manuscript has substantially improved and answered my major and minor points.

2nd Revision - authors' response 05 December 2013

We have now made the corrections that you asked to complement the manuscript.

We have added 5 bullet points to supplement the abstract.

We had exact p-values in the text already, and we have now added these also to the figures/figure legends mentioning the statistical test used.

We have optimized the quality of the blots in Figures 5 and 7. Unfortunately, for some proteins it was impossible to get perfect images, because the original signal was low and thus the exposure time long. A CCD camera was used for signal detection, which for low signals is not as sensitive as the films (e.g. as used in Fig. 5B). However, we believe that the message is clear enough when presented together with the quantifications.
