# Supplementary material for: VEGF-B-induced vascular growth leads to metabolic reprogramming and ischemia resistance in the heart
Source: EMBO Mol Med. 2014 Jan 21;6(3):307–21. doi: 10.1002/emmm.201303147 (PMC3958306; doi:10.1002/emmm.201303147)
Supplement: Supplementary file 10 [file emmm0006-0307-sd10.pdf]

Supporting Information Table 3. Functional annotation clustering of 244 genes upregulated in both VEGF-B TG and AAV-VEGF-B hearts. Analysis was performed using the DAVID bioinformatics resource. The top three clusters are shown with enrichment scores. Count indicates the number of genes in each category and Benjamini controls the false discovery rate.

Annotation Cluster 1	Enrichment Score 4.28	Count	P-value	Benjamini
GOTERM_BP_FAT	vasculature development	15	1.38E-05	0.005
GOTERM_BP_FAT	blood vessel morphogenesis	13	2.41E-05	0.006
GOTERM_BP_FAT	blood vessel development	14	4.43E-05	0.009
GOTERM_BP_FAT	angiogenesis	9	5.53E-04	0.026
**Annotation Cluster 2**	**Enrichment Score 3.84**			
GOTERM_BP_FAT	regulation of calcium ion transport	11	4.74E-08	4.96E-05
GOTERM_BP_FAT	regulation of metal ion transport	11	5.49E-07	3.84E-04
GOTERM_BP_FAT	regulation of ion transport	11	1.89E-06	9.89E-04
**Annotation Cluster 3**	**Enrichment Score 3.64**			
GOTERM_MF_FAT	actin binding	16	5.14E-07	1.97E-04
GOTERM_MF_FAT	cytoskeletal protein binding	20	1.14E-06	2.19E-04
SP_PIR_KEYWORDS	cytoskeleton	19	2.32E-06	5.37E-04
GOTERM_CC_FAT	cytoskeleton	33	2.36E-05	0.002
