# Supplementary material for: VEGF-B-induced vascular growth leads to metabolic reprogramming and ischemia resistance in the heart
Source: EMBO Mol Med. 2014 Jan 21;6(3):307–21. doi: 10.1002/emmm.201303147 (PMC3958306; doi:10.1002/emmm.201303147)
Supplement: Supplementary file 11 [file emmm0006-0307-sd11.pdf]

Supporting Information Table 4. Functional annotation clustering of 40 genes downregulated in both VEGF-B TG and AAV-VEGF-B hearts. The top three clusters are shown with enrichment scores. Count indicates the number of genes in each category, and Benjamini controls the false discovery rate.

**Annotation Cluster 1**	**Enrichment Score 7.25**	**Count**	**P-value**	**Benjamini**
GOTERM_BP_FAT	fatty acid metabolic process	13	9.53E-15	3.72E-12
SP_PIR_KEYWORDS	peroxisome	6	1.08E-06	3.25E-05
GOTERM_CC_FAT	peroxisome	6	4.15E-06	1.41E-04
GOTERM_CC_FAT	microbody	6	4.15E-06	1.41E-04
**Annotation Cluster 2**	**Enrichment Score 5.90**			
GOTERM_BP_FAT	fatty acid metabolic process	13	9.53E-15	3.72E-12
SP_PIR_KEYWORDS	mitochondrion	14	3.64E-10	3.28E-08
GOTERM_CC_FAT	mitochondrion	17	2.91E-08	1.98E-06
SP_PIR_KEYWORDS	transit peptide	9	1.37E-06	3.09E-05
**Annotation Cluster 3**	**Enrichment Score 4.20**			
GOTERM_BP_FAT	fatty acid oxidation	5	2.92E-06	5.70E-04
GOTERM_BP_FAT	lipid oxidation	5	2.92E-06	5.70E-04
GOTERM_BP_FAT	lipid modification	5	1.99E-05	0.003
GOTERM_BP_FAT	fatty acid beta-oxidation	4	3.87E-05	0.004
