# Supplementary material for: VEGF-B-induced vascular growth leads to metabolic reprogramming and ischemia resistance in the heart
Source: EMBO Mol Med. 2014 Jan 21;6(3):307–21. doi: 10.1002/emmm.201303147 (PMC3958306; doi:10.1002/emmm.201303147)
Supplement: Supplementary file 12 [file emmm0006-0307-sd12.pdf]

Supporting Information Table 5. Free long-chain fatty acids in the VEGF-B TG vs. WT hearts. *N*=9+9.

Group	Fatty acid	Fold change (TG vs.WT)	*P*-value	*Q*-value
**Long-chain fatty acid**	nonadecanoate (19:0)	0.75	.0509	.0921
**Long-chain fatty acid**	10-nonadecenoate (19:1n9)	0.79	.0789	.1142
**Long-chain fatty acid**	eicosenoate (20:1n9 or 11)	0.61	< .001	.0061
**Long-chain fatty acid**	dihomo-linoleate (20:2n6)	0.74	.0039	.0256
**Long-chain fatty acid**	adrenate (22:4n6)	0.72	.0021	.0222
**Long-chain fatty acid**	docosadienoate (22:2n6)	0.68	.0046	.0256
**Long-chain fatty acid**	docosatrienoate (22:3n3)	0.71	.0469	.0916
**Essential fatty acid**	docosapentaenoates (n3 DPA)	0.65	.0012	.0135
**Essential fatty acid**	docosahexaenoate (22:6n3)	0.66	< .001	.0061
**Essential fatty acid**	docosapentaenoates (n6 DPA)	0.63	.0028	.0241
**Essential fatty acid**	dihomo-linolenate (20:3n3 or n6)	0.79	.0033	.0256
