# Supplementary material for: VEGF-B-induced vascular growth leads to metabolic reprogramming and ischemia resistance in the heart
Source: EMBO Mol Med. 2014 Jan 21;6(3):307–21. doi: 10.1002/emmm.201303147 (PMC3958306; doi:10.1002/emmm.201303147)
Supplement: Supplementary file 13 [file emmm0006-0307-sd13.pdf]

Supporting Information Methods

VEGF-B KO rats. VEGF-B gene-deleted rats of Sprague-Dawley background were generated using a zinc-finger nuclease based technique by Sigma Advanced Genetic Engineering Labs, Sigma-Aldrich Biotechnology (St. Louis, Missouri, USA) (Cui et al, 2011; Geurts et al, 2009). A 22-base pair segment of exon 1 of the rat VEGF-B gene was replaced with a bacterial lacZ gene with a nuclear localization signal (Sheikh et al, 2008) (a kind gift from Thomas Quertermous) following the endogenous Kozak sequence (Supplemental Fig. 5). Two founder lines were obtained, and both were used for subsequent analyses. The model was verified by sequencing of the inserted lacZ and junctional regions as well as by quantitative PCR and RT-PCR for VEGF-B. The rats were genotyped by PCR of ear DNA with the primers 5'-CCATGGGACTCTGGCTGCC-3' 5'-GGTCTGCTTTCTGACAAACTCG-3' and 5'-CACTTCCGCCAACCTTGAGAG-3', of which the second targets the lacZ insert.

Preparation of the recombinant adeno-associated virus vectors and AAV transduction. Recombinant adeno-associated virus (AAV) vectors (serotype 9) for the expression of human VEGF-B_{167}, VEGF-B_{186} and human serum albumin (HSA) transgenes were constructed as previously described (Bry et al, 2010). AAV particles (300 µl at a concentration of 4.5 x 10^9 virus particles per µl) were injected via a tail vein to Wistar rats. For the male rats, the injection was repeated 10 weeks later. Samples were taken after four (male rats) or two (female rats) months of transgene expression. An additional experiment was performed where VEGF-B was expressed for two weeks.

Blocking VEGF-VEGFR2 signaling with soluble VEGFR-2 *in vivo*. 20 C57Bl/6J mice (5 per group) were injected intraperitoneally with either AAV9-Empty (empty vector), AAV9-mVEGF-B186 + Empty, AAV9-mVEGFR-2(D1-7) + Empty or AAV9-mVEGF-B + AAV9-mVEGFR-2(D1-7) to achieve a similar viral load in all groups (7.0 x 10^{11} virus particles per mouse). After four weeks of expression, the sera and hearts were collected for the analyses.

NOS blocking experiment *in vivo*. 30 C57Bl/6J mice (5 per group) of which 10 were eNOS-/- mice (JAX laboratories), were injected intraperitoneally with either AAV9-control vector or AAV9-mVEGF-B186 (7.0 x 10^{11} virus particles per mouse). Ten mice (5 control and 5 VEGF-B) were given L-NAME (0.5g/L) in their drinking water during the whole experiment. Four weeks after the injections, the sera and hearts were collected for analysis.

Semithin serial sectioning and transmission electron microscopy. Six WT and six TG hearts were harvested and fixed in 2.5% (v/v) glutaraldehyde. The hearts were then post-fixed in 1% OsO_4, dehydrated in ethanol, and embedded in epoxy resin. Hearts were separated about 2 mm distal and parallel to the coronary sulcus. Numerous semithin and ultrathin sections were obtained from the left and right ventricle, and from the septum. Sections (80–90 nm thick) were mounted on copper grids coated with polyvinyl formal (Formvar; Fluka, Buchs, Switzerland), stained with lead citrate and uranyl acetate, and viewed with a Philips EM-400 model transmission electron microscope (Djonov et al, 2000).

Microvascular diameter measurements. 2120 microvessels from the WT and 2560 from the TG group were measured taking the smallest possible diameter. Subendocardial (inner 1/3 of the myocardium) vessels were compared with the subepicardial region (outer 1/3 of the myocardium).

Vascular casting. Vascular casts from 6 WT and 8 TG animals were prepared using a standard procedure previously described (Djonov et al, 2000). Briefly, the heart vasculature was perfused with a freshly prepared solution of Mercox® (Vilene Company, Japan) containing 0.1 mL of accelerator per 5 mL of resin. The samples were then coated with gold to a thickness of 10 nm and viewed in a Philips XL-30 SFEG scanning electron microscope.

Echocardiography of 20-22-month old rats. Transthoracic echocardiography was performed under isoflurane anesthesia with an Acuson Sequoia 512 Ultrasound System and an Acuson Linear 15L8 14 MHz transducer (Siemens Medical Solutions, Mountain View, CA, USA).

Maximal exercise capacity. Rats were first adapted to treadmill running on three separate days before maximal running capacity was tested three times. The rats ran first at 9, 12 and 15 m/min each for 5 min, after which the velocity was increased every two minutes until the rats were unable to continue. Maximal oxygen consumption and carbon dioxide production were measured continuously during the test.

Clinical chemistry from serum samples. Serum samples from TG and AAV-rats were analyzed for 20 clinical chemistry parameters (S-K, S-Na, S-Cl, S-ALT, S-ALP, S-Crea, S-Glu, S-Prot, S-ALB, S-HDL, S-LDL, S-Trig, S-CK, S-AST, S-GT, S-Urea, S-Pi, S-Bil, S-CA, S-Chol) using routine clinical laboratory techniques. Free fatty acid levels were measured with NEFA R2 kit downscaled to microplate format (Wako).

Histochemical analysis of the infarcted hearts. Infarct sizes were estimated from Masson's trichrome stained images (Pfeffer et al, 1979; Zentilin et al, 2010). Paraffin sections from MI hearts were stained with FITC-conjugated lectin (FL1171, Vector Laboratories) and mouse anti-SMA (Cy3-conjugated, clone 1A4, Sigma). In addition to vascular quantification, semi-quantitative analysis of SMA-positive myofibroblasts in scar tissue was performed by assessing the number of heart sections per genotype containing no, few, or numerous myofibroblasts.

Assessment of myocardial perfusion, infarct size and oxygen consumption with positron emission tomography (PET). The rats (11 TG, 17 WT) were imaged with a small animal PET scanner (Inveon or DPET, Siemens, Knoxville, TN, USA) 4 weeks after coronary occlusion. 45 ± 11 MBq of 11C-acetate was injected to the rat teil vein in 0.4 – 1.0 ml over 10 seconds. Images were acquired in 3D for 10 minutes and stored in listmode format.

Myocardial blood flow was calculated from the 11C-acetate images by using the single compartment model and the resulting rate constant K_1 (1/min) values of the LV myocardium were displayed as a polar map. Each polar map was normalized to its own maximum. Then, myocardial infarct size was measured as the fraction of polar map elements with K_1 values less than 60 % of the maximum uptake and expressed as percentage of the total LV. In order to study regional myocardial perfusion, the polar maps were also analysed using the AHA 17 segment model. Myocardial oxygen consumption was assessed by applying monoexponential fitting to calculate 11C-acetate clearance rate (K_{mono}) in the segments that were remote from the infracted area (septum and inferior wall).

In order to validate measurement of myocardial infarct size by 11C-acetate, a subgroup of the rats (*N*=16) was injected with 40 ± 5 MBq of 18F-FDG, a marker of myocardial glucose metabolism and viability, in a separate imaging session. Images were reconstructed as described above and myocardial infarct size was determined as the fraction of polar map elements with tracer uptake less than 60 % of the maximum 18F-FDG uptake. In these rats, myocardial infarct size measured by 11C-acetate perfusion and 18F-FDG uptake showed the best correlation (r=0.87, R^2=0.75, P<.01) when 60% threshold was used for 11C-acetate perfusion.

Immunofluorescence and immunohistochemistry. The antibodies used for immunostaining were: mouse anti-SMA (Cy3-conjugated, clone 1A4, C6189, Sigma), mouse anti-rat RECA-1 (MCA970, Serotec), goat anti-human VEGF-B (AF751, R&D Systems), mouse anti-human dystrophin-2 (clone Dy8/6C5, Novocastra), mouse anti-rat CD45 (cat #550566, BD Biosciences), mouse anti-rat ED1 (MCA341R, Serotec), rat anti-mouse VEGFR-1 (5B12, ImClone) and goat anti-mouse VEGFR-2 (AF644, R&D Systems). Alexa Fluor 488- and 594-conjugated secondary antibodies (Molecular Probes) were used for detection. Beta-galactosidase activity was detected as previously described (Shalaby et al, 1995). For immunohistochemistry, antibodies against phosphorylated rpS6 at $Ser^{235/236}$ and Erk1/2 MAPK at Thr^{202}/Tyr^{204} (Cell Signaling) and Cyclin D1 (RM-9104-5, Thermo Scientific) were used together with the tyramide signal amplification kit (NEL700001KT, Perkin Elmer) and AEC detection. Sections were counterstained with hemalum.

Western blotting. VEGF-B expression was confirmed by Western blotting of heart lysates with an antibody against $VEGF\text{-}B_{167/186}$ (AF751, R&D). In phosphorylation experiments, antibodies recognized phosphorylated Akt at Thr^{308} and Ser^{473}, rpS6 at $Ser^{235/236}$ and $Ser^{240/244}$,

p38 MAPK at Thr^{180}/Tyr^{182}, Erk1/2 MAPK at Thr^{202}/Tyr^{204}, S6K1 at Thr^{389}, 4EBP1 at $Thr^{37/46}$, AMPK at Thr^{172} and VEGFR-2 at Tyr^{1175} (Cell Signaling). Moreover, total S6K1, (Santa Cruz Biotechnology), rpS6, Akt, Erk 1/2, p38, AMPK, (Cell Signaling), VEGFR-2 (AF644, R&D), were analyzed using specific antibodies by re-probing the membrane after blotting for respective phospho-antibodies. Antibodies against cytochrome c (Santa Cruz Biotechnology), fatty acid synthase (Cell Signaling), perilipin5 (Progen), PDK4 (Novus Biologicals) and PGC-1α (Calbiochem) were also used. Total protein content was determined using the BCA protein assay (Pierce Biotechnology). Proteins were visualized by ECL (Pierce Biotechnology) and quantified (band intensity × volume) using ChemiDoc XRS in combination with Quantity One software (Bio-Rad Laboratories). The uniformity of protein loading was confirmed with Ponceau S staining and by re-probing the membranes with an antibody against GAPDH (Abcam).

qPCR. Total RNA was isolated from the left ventricle with TRIsure reagent (Bioline, Luckenwalde, Germany) and further purified with NucleoSpin RNA II (Macherey-Nagel). RNA was transcribed to cDNA using iScript kit (Bio-Rad) and qPCR was carried out following standard procedures using SYBR green or TaqMan primer-probe sets (Supplemental Table 6). All data were normalized to 18S, β-actin, and TBP housekeeping genes (Hprt1 in human samples), and quantification was performed using the $2^{-\Delta\Delta CT}$ method.

Microarray analysis. RNA samples from TG vs. WT rats and AAV-VEGF-B versus AAV-HSA rats (*N*=6 in all groups) were analyzed with the genome-wide Illumina RatRef-12 Expression BeadChip (BD-27-303; Illumina Inc.). Illumina's GenomeStudio software was used for initial data analysis and quality control. For the two-week AAV-experiment, Affymetrix Rat Gene 2.0 chips were used, since the production of Illumina chips was

discontinued. For all experiments, detailed data analyses were performed with the Chipster software (www.chipster.csc.fi) (Kallio et al, 2011). After quantile normalization, statistically significant differences in individual genes between the groups were tested using Empirical Bayes statistics and the Benjamin-Hochberg algorithm controlling false discovery rate (FDR). Adjusted FDR values of $P<0.05$ were considered significant. The gene array data have been deposited in the Gene Expression Omnibus, accession number GSE38457.

Gene Functional Classification Analysis. The significantly changed transcriptomes were uploaded to the DAVID Bioinformatics Resource where the Functional Annotation Clustering tool was used to generate clusters of overrepresented Gene Ontology terms (Huang da et al, 2009a; Huang da et al, 2009b). Gene set enrichment analysis (GSEA, http://www.broadinstitute.org/gsea) was performed for the normalized unfiltered data set (Mootha et al, 2003; Subramanian et al, 2005).

Metabolomics. Samples from left ventricle were extracted and prepared for analysis using Metabolon's (Metabolon Inc.) standard solvent extraction method. The extracted samples were split into equal parts for analysis on the GC/MS and LC/MS/MS platforms. The present dataset comprises a total of 315 named biochemicals. Following log transformation, imputation with minimum observed values for each compound and normalization to Bradford protein, Welch's two-sample *t*-test was used to identify biochemicals that differed significantly between experimental groups. An estimate of the false discovery rate (*q*-value) was calculated to take into account the multiple comparisons.

Measurement of malonyl-CoA levels. Detection and quantification of malonyl-CoA esters was accomplished by extracting CoA esters from powdered tissue and measuring with a

modified HPLC. Peaks corresponding to malonyl-CoA and acetyl-CoA were integrated by the Beckman System Gold software package. These analyses were done at Sanford-Burnham Institute, Orlando.

Quantification of cardiac lipids. Lipids were extracted using chloroform-methanol (2:1) with minor changes to the classic Folch method (Folch et al, 1957). Heart tissue (20 – 30 mg) was solubilized in 1 ml chloroform-methanol utilizing TissueRuptor (Qiagen) in glass tubes. After solubilization, 2 ml chloroform and 3 ml of 0.9 % NaCl solution was added to the homogenate and mixed by vortexing for 3 - 5 seconds. Samples were centrifuged for 3 minutes at 2000g to separate the organic (lower) and aqueous (upper) phases. Lower phase was carefully collected using a glass Pasteur pipet and another 2 ml chloroform was added and the procedure repeated. Chloroform was evaporated in a clean glass tube under nitrogen and lipids were solubilized in PBS, 1% Tx100 by overnight mixing at 37^0C. Triglycerides, total cholesterol and phospholipids were measured by colorimetric reactions utilizing specific quantification kits (Cobas, Roche/Hitachi).

Angiotensin II treatment. Twenty-three C57BL/6J mice were subcutaneously implanted with Alzet osmotic minipumps (Alzet model 1002, Durect Corporation, Cupertino, CA). 12 mice received Angiotensin II (0.1 mg/kg/h) and 11 mice NaCl for 14 days. Hearts were collected, RNA extracted similarly to the rat hearts, and analyzed for VEGF-B mRNA expression.

Primers used in QPCR.

Primer	Sequence	Ref.
**rGATA4 fwd**	TGGGTGCAGTGCAGATGTTAC	(Bostrom et al, 2010)
**rGATA4 rev**	ATGTTCAGGCTGGAGAGCAAG	(Bostrom et al, 2010)
**rNkx2.5 fwd**	CTCGGATTTCACACCCACACT	(Bostrom et al, 2010)
**rNkx2.5 rev**	CTCCGGGTCCTGATATGGAAT	(Bostrom et al, 2010)
**rCited4 fwd**	ACGAGGGTGGTTTTGCAGTCT	(Bostrom et al, 2010)
**rCited4 rev**	CAACTCAGCCAGACAGAGGAA	(Bostrom et al, 2010)
**rCyclin D1 fwd**	TGGAGCCCCTGAAGAAGAG	(Bostrom et al, 2010)
**rCyclinD1 rev**	AAGTGCGTTGTGCGGTAGC	(Bostrom et al, 2010)
**rC/EBPβ fwd**	GGGGTTGTTGCTGTTGATGT	(Bostrom et al, 2010)
**rC/EBPβ rev**	GCTCGAAACGGAAAAGGTTC	(Bostrom et al, 2010)
**rDll4 fwd**	TGCAATGAATGTATCCCCCA	
**rDll4 rev**	CTTGCACGGAGAGTGGTGAG	
**rJag1**	Taqman probes	
**rNotch4 fwd**	AGGAGGAAGAAGGGCGGTAG	
**rNotch4 rev**	GGGATCCTCCACACAGAAG	
**rNotch1**	Taqman probes	
**rS18 fwd**	TCAAGAACGAAAGTCGGAGG	
**rS18 rev**	GGACATCTAAGGGCATCAC	
**rBeta-actin fwd**	GGAAAAGAGCCTCAGGGCAT	
**rBeta-actin rev**	GAAGAGCTATGAGCTGCCTGA	
**rTBP fwd**	TGCACAGGAGCCAAGAGTGAA	
**rTBP rev**	CACATCACAGCTCCCCACCA	
**mDll4 fwd**	TGCAATGAATGTATCCCCCA	
**mDll4 rev**	CTTGCACGGAGAGTGGTGAG	
**mTBP fwd**	GAAGCTGCGGTACAATTCCAG	
**mTBP rev**	CCCCTTGTACCCTTCACCAAT	
**mVegfb fwd**	AGCCACCAGAAGAAAGTGGT + Taqman probes	
**mVegfb rev**	GCTGGGCACTAGTTGTTTGA	
**hVEGFB fwd**	GAAAGTGGTGTCATGGATAG	
**hVEGFB rev**	ATGAGCTCCACAGTCAAG	
**hHPRT1 fwd**	TGAGGATTTGGAAAGGGTGT	
**hHPRT1 rev**	TCCCCTGTTGACTGGTCATT	
