# Supplementary material for: VEGF-B-induced vascular growth leads to metabolic reprogramming and ischemia resistance in the heart
Source: EMBO Mol Med. 2014 Jan 21;6(3):307–21. doi: 10.1002/emmm.201303147 (PMC3958306; doi:10.1002/emmm.201303147)
Supplement: Supplementary file 14 [file emmm0006-0307-sd14.pdf]

References

Bostrom P, Mann N, Wu J, Quintero PA, Plovie ER, Panakova D, Gupta RK, Xiao C, MacRae CA, Rosenzweig A et al (2010) C/EBPbeta controls exercise-induced cardiac growth and protects against pathological cardiac remodeling. Cell 143**:** 1072-1083

Bry M, Kivela R, Holopainen T, Anisimov A, Tammela T, Soronen J, Silvola J, Saraste A, Jeltsch M, Korpisalo P et al (2010) Vascular endothelial growth factor-B acts as a coronary growth factor in transgenic rats without inducing angiogenesis, vascular leak, or inflammation. Circulation 122**:** 1725-1733

Cui X, Ji D, Fisher DA, Wu Y, Briner DM, Weinstein EJ (2011) Targeted integration in rat and mouse embryos with zinc-finger nucleases. Nat Biotechnol 29**:** 64-67

Djonov V, Schmid M, Tschanz SA, Burri PH (2000) Intussusceptive angiogenesis: its role in embryonic vascular network formation. Circ Res 86**:** 286-292

Folch J, Lees M, Sloane Stanley GH (1957) A simple method for the isolation and purification of total lipides from animal tissues. J Biol Chem 226**:** 497-509

Geurts AM, Cost GJ, Freyvert Y, Zeitler B, Miller JC, Choi VM, Jenkins SS, Wood A, Cui X, Meng X et al (2009) Knockout rats via embryo microinjection of zinc-finger nucleases. Science 325**:** 433

Heather LC, Carr CA, Stuckey DJ, Pope S, Morten KJ, Carter EE, Edwards LM, Clarke K (2010) Critical role of complex III in the early metabolic changes following myocardial infarction. Cardiovasc Res 85**:** 127-136

Huang da W, Sherman BT, Lempicki RA (2009a) Bioinformatics enrichment tools: paths toward the comprehensive functional analysis of large gene lists. Nucleic Acids Res 37**:** 1-13

Huang da W, Sherman BT, Lempicki RA (2009b) Systematic and integrative analysis of large gene lists using DAVID bioinformatics resources. Nat Protoc 4**:** 44-57

Kallio MA, Tuimala JT, Hupponen T, Klemela P, Gentile M, Scheinin I, Koski M, Kaki J, Korpelainen EI (2011) Chipster: user-friendly analysis software for microarray and other high-throughput data. BMC Genomics 12**:** 507

Mootha VK, Lindgren CM, Eriksson KF, Subramanian A, Sihag S, Lehar J, Puigserver P, Carlsson E, Ridderstrale M, Laurila E et al (2003) PGC-1alpha-responsive genes involved in oxidative phosphorylation are coordinately downregulated in human diabetes. Nat Genet 34**:** 267-273

Pfeffer MA, Pfeffer JM, Fishbein MC, Fletcher PJ, Spadaro J, Kloner RA, Braunwald E (1979) Myocardial infarct size and ventricular function in rats. Circ Res 44**:** 503-512

Shalaby F, Rossant J, Yamaguchi TP, Gertsenstein M, Wu XF, Breitman ML, Schuh AC (1995) Failure of blood-island formation and vasculogenesis in Flk-1-deficient mice. Nature 376**:** 62-66

Sheikh AY, Chun HJ, Glassford AJ, Kundu RK, Kutschka I, Ardigo D, Hendry SL, Wagner RA, Chen MM, Ali ZA et al (2008) In vivo genetic profiling and cellular localization of apelin reveals a hypoxia-sensitive, endothelial-centered pathway activated in ischemic heart failure. Am J Physiol Heart Circ Physiol 294**:** H88-98

Subramanian A, Tamayo P, Mootha VK, Mukherjee S, Ebert BL, Gillette MA, Paulovich A, Pomeroy SL, Golub TR, Lander ES et al (2005) Gene set enrichment analysis: a knowledge-based approach for interpreting genome-wide expression profiles. Proc Natl Acad Sci USA 102**:** 15545-15550

Zentilin L, Puligadda U, Lionetti V, Zacchigna S, Collesi C, Pattarini L, Ruozi G, Camporesi S, Sinagra G, Pepe M et al (2010) Cardiomyocyte VEGFR-1 activation by VEGF-B induces compensatory hypertrophy and preserves cardiac function after myocardial infarction. FASEB J 24**:** 1467-1478
