# Supplementary material for: VEGF-B-induced vascular growth leads to metabolic reprogramming and ischemia resistance in the heart
Source: EMBO Mol Med. 2014 Jan 21;6(3):307–21. doi: 10.1002/emmm.201303147 (PMC3958306; doi:10.1002/emmm.201303147)
Supplement: Supplementary file 15 [file emmm0006-0307-sd15.pdf]

VEGF-B-induced vascular growth leads to metabolic reprogramming and ischemia resistance in the heart

Kivelä et al.

Supporting Information

Table of Content

Supporting Information Figures 1-6

Supporting Information Tables 1-5 (Table 2 as separate Excel-file Dataset)

Supporting Information Materials and Methods

Supporting Information References
